# Artificial Intelligence in Nephrology: From Early Detection to Clinical Management of Kidney Diseases

**DOI:** 10.3390/bioengineering12101069

**Published:** 2025-10-01

**Authors:** Alessia Nicosia, Nunzio Cancilla, José David Martín Guerrero, Ilenia Tinnirello, Andrea Cipollina

**Affiliations:** 1 Department of Engineering, Università degli Studi di Palermo, Viale delle Scienze Ed. 6, 90128 Palermo, Italy; alessia.nicosia@unipa.it (A.N.); nunzio.cancilla@unipa.it (N.C.); ilenia.tinnirello@unipa.it (I.T.); 2 Department of Electronic Engineering, Escola Tècnica Superior d’Enginyeria, Universitat de València (ETSE-UV), Despatx 3.2.26 Avgda. Universitat, s/n., 46100 València, Spain; jose.d.martin@uv.es; 3 Valencian Graduate School and Research Network of Artificial Intelligence (ValgrAI), 46010 València, Spain

**Keywords:** nephrology AI, machine learning dialysis, kidney disease, hemodialysis, prediction, detection, renal replacement therapy optimization

## Abstract

Artificial Intelligence (AI) is transforming the healthcare field, offering innovative tools for improving the prediction, detection, and management of diseases. In nephrology, AI holds the potential to improve the diagnosis and treatment of kidney diseases, as well as the optimization of renal replacement therapies. In this review, a comprehensive analysis of recent literature works on artificial intelligence applied to nephrology is presented. Two key research areas structure this review. The first section examines AI models used to support early prediction of acute and chronic kidney disease. The second section explores artificial intelligence applications for hemodialytic therapies in renal insufficiency. Most studies reported high accuracy (e.g., accuracy ≥ 90%) in early prediction of kidney diseases, while fewer addressed therapy optimization and complication prevention, typically reporting moderate-to-high performance (e.g., accuracy ≃ 85%). Filling this gap and developing more accessible AI solutions that address all stages of kidney disease would therefore be crucial to support physicians’ decision-making and improve patient care.

## 1. Introduction

### 1.1. Artificial Intelligence

Artificial Intelligence is a discipline that focuses on creating an intelligent system able to perform tasks that require human intelligence, such as reasoning, planning, and natural language understanding, through the use of algorithms and logical models, without needing to learn from data [1].

AI uses a variety of techniques and approaches to achieve these capabilities. Some systems rely on simple rules and logic, while others use more advanced algorithms that allow them to learn and adapt over time. This versatility allows AI to be applied in many different domains and industries, making it a powerful tool for improving productivity and innovation.

There are, in fact, several subcategories of artificial intelligence, each capable of using a different method to achieve intelligent behavior [2], and the main ones are the following.

Machine Learning (ML) focuses specifically on the ability of machines to learn from data and improve their performance over time without being explicitly programmed.

Rules-based Systems use predefined rules to make decisions or solve problems. They operate based on specific logic and are often used in expert systems to mimic human decision-making in specific domains.

Natural Language Processing (NLP) focuses on enabling machines to understand, interpret, and respond to human language. Applications include chatbots, translation services, and voice recognition systems.

Computer Vision allows computers to interpret and understand visual information from the world, such as images and videos. It is used in applications like facial recognition, autonomous vehicles, and medical imaging.

Robotics involves creating intelligent machines that can perform tasks in the physical world. This includes everything from industrial robots to drones and service robots.

Planning and Decision-Making focuses on developing algorithms that help systems make decisions based on available information. This includes optimization problems, game theory, and multi-agent systems.

Expert Systems are computer programs that simulate the judgment and behavior of a human or an organization with expertise in a particular area. They are designed to solve complex problems by reasoning through bodies of knowledge.

Fuzzy Logic Systems allow for degrees of truth unlike traditional binary sets (true/false). This approach is useful in systems that require a more nuanced understanding of data, like control systems in appliances.

### 1.2. Machine Learning and Deep Learning

The main subcategory of Artificial Intelligence is Machine Learning, which is based on the concept of self-learning.

To implement an ML model successfully, three key components are required: a training dataset, a learning model capable of approximating the behavior of the system to be predicted, and a validation and/or testing dataset.

Specifically, an ML model is a data learning system. To train the model, a dataset containing examples of the problem to be solved must be provided. The model tries to understand the relationships among the data and modifies its parameters so that its predictions align as closely as possible with the training data.

Once trained, the model can be used to make predictions on new data. The effectiveness of a model is evaluated using new validation or test data, which are not used during training. This leads to the creation of very efficient tools capable of making predictions or decisions on new data, often faster and more accurately than traditional models, which often present limitations on particularly complex systems.

Among the most promising models used in ML, deep Artificial Neural Networks (ANNs) have given rise to a further subdiscipline called Deep Learning (DL). An artificial neural network is an adaptive system that learns by using at least two layers of interconnected nodes, which mimic the human neurons. The strength of these interactions is determined by their so-called “weights”. The greater the weight, the more important the contribution of that connection to the output. An important component of the learning process in an artificial neural network is gradient descent. This algorithm optimizes the weights of the connections between nodes during the training process. Essentially, gradient descent seeks to minimize the error between the network’s predictions and the desired outcomes by iteratively updating the weights based on the direction and magnitude of the gradient of the cost function. The cost function quantifies the difference between the predicted outputs of the network and the actual target values, serving as a measure of how well the model is performing. In practice, gradient descent allows the ANN to “learn” from data: starting from randomly initialized weights, the model progressively adapts, improving its performance in recognizing patterns, classifying data, and predicting future events without requiring human intervention [3].

Typical non-strictly deep ANNs are Bayesian Neural Networks (BNNs), machine learning models that, instead of having fixed weights, represent the weights as probabilistic distributions, making it possible to manage and quantify uncertainty in situations where it is an important factor, such as in medicine, robotics, and finance [4]. If ANNs contain at least three layers, they can be classified as “deep”, capable of modeling more complex relationships. In the rest of this paper, we will use the term “Neural Networks” (NNs) to refer to networks belonging to the “deep” field, and the global name of AI to collectively denote artificial intelligence, machine learning, and deep learning, despite the differences between all these approaches.

Since the last century, the use of artificial intelligence techniques has interested many areas, ranging from business to tourism, from telecommunications to health care [5,6,7,8].

Specifically, the use of AI in healthcare dates back to the 1960s, when the growing potential offered by advanced computational techniques made it possible to automate the interpretation of ElectroCardioGrams (ECGs) for the diagnosis of cardiovascular diseases [6]. Despite the initial enthusiasm, the use of artificial intelligence tools in the medical field remained “dormant” until the 1970s, when Gagliardo et al. [7] applied multilinear regression to predict cell-mediated immune reactivity to tumor antigens [8] using a dataset previously analyzed by Heppner et al. [9]. Early expert systems were also developed, including PIP, which modeled disease states in edema patients [10], and MYCIN, designed to suggest antibiotic therapy for bacteremia [8].

All these applications showed the benefit that could be derived from an accurate model that allows for predicting the result even in circumstances in which a particular experiment is not performed, as in the second case, or to stimulate further refinement of the evolving cognitive theory on a given pathology, as in the last two.

It is important to emphasize that, beyond technical performance, the successful integration of AI into clinical workflows requires addressing key issues such as algorithm transparency, data privacy, fairness, and ethical responsibility. These aspects are crucial to ensure patient safety, build clinicians’ trust, comply with regulatory standards, and achieve real clinical impact.

It is encouraging that researchers have devoted increasing attention to these issues in recent years, promoting the growing use of artificial intelligence techniques in the biomedical field to the present day. An example is the increasing use of AI in the study of kidney disease and its application to renal replacement therapies.

### 1.3. Kidney Diseases and Treatments

Kidney Diseases (KDs) can be divided into two main groups:

(i). Acute Kidney Injury (AKI) refers to a kidney malfunction characterized by rapid deterioration of its functions. Generally, AKI causes renal dysfunction in patients already suffering from other diseases, only for short periods. Despite its reversibility, an incomplete recovery can lead to the terminal stages of the disease and, though not commonly, to death.
(ii). Chronic Kidney Disease (CKD) refers to sustained kidney malfunction related to the presence of structural or functional abnormalities. CKD is stated when a reduction in the so-called “estimated Glomerular Filtration Rate” (eGFR) lasts for three months or more [11]. Chronic kidney disease is very common in patients with diabetes and hypertension and can lead to death. Specifically, eGFR is the best index for kidney function as it measures the kidneys’ ability to filter toxins and waste from the blood. Dialysis treatment or a kidney transplant is required when eGFR is very low. Equation (1) shows the Cockcroft–Gault law for the eGFR calculation [12]:

(1) $$eGFR=\frac{\left(140-age\right)\cdot weight\left(kg\right)\cdot 0.85(if\;female)}{72\cdot serum\;creatinine\;(mg/dL)}$$

where *serum creatinine* is a waste product present in the blood and resulting from muscle activity, which is normally excreted with urine.

Chronic kidney disease is one of the leading causes of death in the 21st century, since to date it affects more than 800 million individuals worldwide, with a significant increase in deaths over the past two decades [13]. Despite receiving treatment, CKD patients often experience overlapping episodes of AKI, as this condition is frequently triggered by the use of medication or the presence of previous diseases [14]. Therefore, early detection of chronic kidney disease and its prevention can be crucial to avoid further complications.

**Figure 1:**
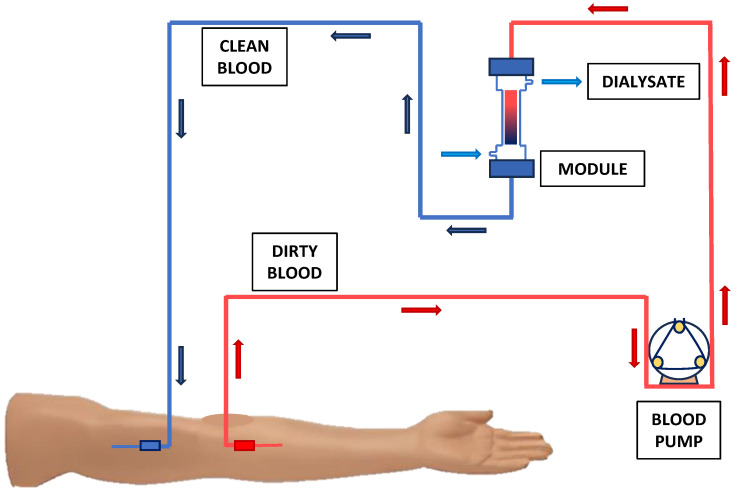
Schematic of the HemoDialysis (HD) circuit.

There are five levels of CKD, the last of which is called “End-Stage Kidney Disease” (ESKD). The state of progressive loss of kidney function caused by this condition results in the need for renal replacement therapy, such as HemoDialysis (HD) or transplantation, to ensure patients’ survival. Specifically, standard hemodialysis is a process based on the use of a semi-permeable membrane with a hollow fiber configuration. The solute removal through the membrane takes place by means of a diffusive mechanism from the blood to a dilute rinsing solution, called dialysate [15]. The driving force for the separation is the difference in concentration between the two compartments. The fibers are contained within a cylindrical shell housing. It constitutes the core unit of the treatment and contains four ports, two for the dialysate and two for the blood. To improve mass transfer efficiency, the two fluids flow in the module in countercurrent mode. The module, thanks to a potting system, is able to guarantee complete segregation between the two fluids, which is essential to avoid contamination problems. In particular, blood is taken from the patient through a vascular access generally created in the arm, pressurized by a pump, and sent to the module. After the removal of toxins and other waste products, the purified blood returns to the patient through a second vascular access. Figure 1 shows the basic aspects of the hemodialysis process.

Variants of hemodialysis treatment are HemoFiltration (HF), HemoDiaFiltration (HDF), and Peritoneal Dialysis (PD).

Hemofiltration is a dialysis technique based on the removal of uremic toxins exclusively by convection. Unlike standard HD, in HF the main driving force is not a concentration gradient but a pressure gradient, which forces the passage of part of the flowing blood (plasmatic components) through the porous membrane and removes plasmatic toxins, entraining and removing also medium and high molecular weight solutes [16]. To maintain a constant blood volume and provide electrolytes, hemofiltration requires the addition of replacement fluids before or after the blood passes through the module. In the former case, it is referred to as pre-dilution replacement, and, in the latter, as post-dilution.

Hemodiafiltration is a renal therapy that combines the characteristics of hemodialysis and hemofiltration. This process combines diffusion and convection, allowing the removal of excess fluids and toxic solutes of low, medium, and high molecular weight, thus best simulating natural renal filtration. This offers some advantages over traditional blood purification techniques, including a more efficient removal of toxins, a better control of water balance, and a greater elimination of certain molecules. This makes hemodiafiltration the most used technique.

**Figure 2:**
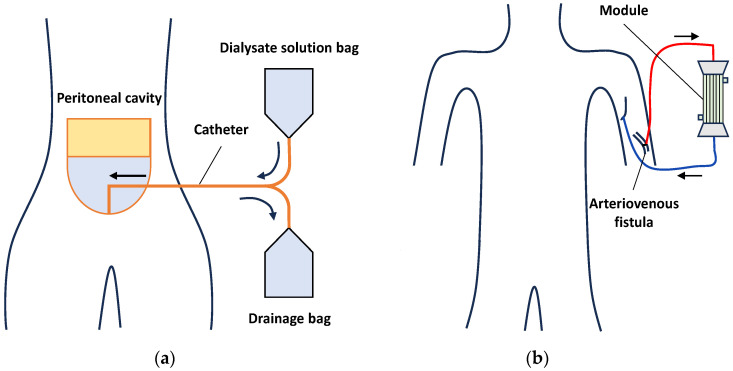
Graphic representation of Peritoneal Dialysis (PD) (**a**) and classic HemoDialysis (HD) (**b**). In (**a**), the peritoneum in the patient’s peritoneal cavity is used as the membrane through which excess fluid and solutes are removed from the blood to the dialysate. In (**b**), the module is used to purify the patient’s blood, drawn via an ArterioVenous Fistula (AVF).

**Figure 3:**
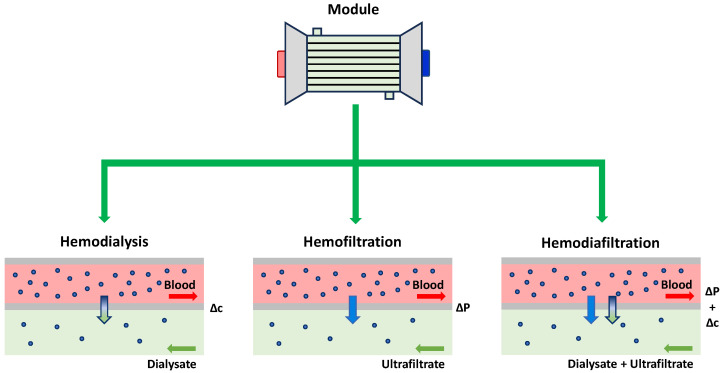
Scheme of the three hemodialytic variants. Solute clearance can be achieved by diffusion or convection. In HemoDialysis (HD), solutes move down their concentration gradient (Δc) from the blood to the dialysate. In HemoFiltration (HF), a pressure gradient (ΔP) forces solutes and fluids across the filter. In HemoDiaFiltration (HDF), both Δc and ΔP act to remove solutes and fluids across the filter.

Peritoneal dialysis offers the advantage of being feasible for home-based renal treatment. During peritoneal dialysis, a cleansing fluid flows through a tube into the abdomen of the patient [17]. The inner lining of the abdomen, known as the peritoneum, acts as a filter and removes waste metabolites, toxins, etc., from the blood. After a certain period of time, the liquid with accumulated waste flows out of the abdomen and is thrown away. This therapy offers the benefit of being applicable to patients with chronic kidney disease who are not eligible for a kidney transplant or hemodialysis. However, it may not be suitable for patients experiencing abdominal discomfort, drainage problems, or peritoneal membrane complications. Therefore, it is necessary for the patient’s medical team to conduct a comprehensive assessment to determine the most suitable therapeutic approach from the available options. Figure 2 shows schematically the main differences between peritoneal dialysis (Figure 2a) and classic hemodialytic treatment (Figure 2b), while Figure 3 shows a specific focus on the three possible hemodialytic variants.

### 1.4. Artificial Intelligence Application on Kidney Diseases and Hemodialysis

Over the years, the use of AI has shown increasing potential in this regard; so much so that its use is now a valuable decision support for experts in many medical fields, including renal care.

AI tools have influenced the field of nephrology since the 1980s, particularly in areas where disease prevention or detection significantly impacts patient survival.

In 1987, Gan et al. [18] described the development of RENPAD, an interactive computer program designed to assist the physician in the preliminary diagnosis of primary renal disease using a rule-based system, OPS5, which allowed for faster diagnostic sessions. It is important to emphasize the relevance of this first example of application of artificial intelligence tools in the renal field, because it later inspired other researchers to invest in this area.

Indeed, in 1992, Agar et al. [19] described in their study a non-invasive computational technique for the assessment and interpretation of clinical and laboratory data in glomerular disease, with the aim of avoiding renal biopsy. While the accuracy was not particularly high, this model can be regarded as one of the first attempts aimed at reducing both the cost and the morbidity associated with investigating glomerular disease in cases where renal biopsy is deemed dangerous or contraindicated.

Then, in 1993, Chang et al. [20] developed a Renal Mass Diagnostic System (RMDS) by using ILIAD, an expert system created for diagnostic consultations. Seventy-two cases of renal mass have been tested on this system, obtaining a diagnostic accuracy of 75%. This system was also able to show the cost of the various possible diagnostic procedures, so that the user could choose the most convenient one to confirm the ILIAD diagnosis. Also in 1993, McMichael et al. [21] realized a very interesting Intelligent Dosing System (IDS), capable of predicting the appropriate doses of the immunosuppressive drug FK506 to be administered to kidney or liver transplant patients. The good performance of the IDS provided a first example of a model capable of minimizing the pharmaceutical dose administered to immunosuppressed patients in order to reduce potential toxicity, costs, and duration of hospitalization.

In 1992, Gray et al. [22] even tried to apply such computerized systems in the context of dialysis. They developed an AI tool capable of alerting doctors and nurses at an early stage to potential abnormal interactions between dialysis patients and prescribed drug therapies. This monitoring system was created with the aim of ensuring that drug therapy was consistent and correct, according to the rules created by healthcare providers, thus avoiding occasional errors in drug therapy.

Another interesting application was developed in 2001 by Akl et al. [23], who implemented a neural network model to study and predict concentrations of urea during a hemodialysis session. After the training, the NN model was able to predict hemodialysis session time needed to reach a target Solute Removal Index (SRI) in patients not previously studied by the NN model with an acceptable prediction error of 10.90%. Also, in 2001, Fernàndez et al. [24] implemented a supervised NN to predict the equilibrated post-dialysis blood Urea (eqU) at 60 min after the end of the hemodialysis session, essential for determining the equilibrated Kt/V (parameter used to evaluate the adequacy of a dialysis treatment), a major determinant of morbidity and mortality in hemodialysis patients. Similarly, in 2003, Ray et al. [25] used Radial Basis Function Neural Network (RBFNN) and Generalised Regression Neural Network (GRNN), functional models even on a small dataset, to predict the Kt/V of a dialysis session and, thus, the rate of urea removal during the treatment. The use of the models described in references [24,25] proved in both cases to be better than traditional methods, demonstrating once again the great potential of artificial intelligence tools in aiding doctors and nurses.

In recent years, applications in the renal field are growing further, especially to prevent or identify the onset of disease, kidney size, complications of previous diseases, or complications from the hemodialysis process. The great interest in this field has led research to increasingly better results, such that in many cases they have supplanted previous ones. For example, in 2015, Hussain et al. [26] proposed a supervised learning approach for kidney volume estimation starting from 3D Computational Tomography (CT) images and using a regression model to simultaneously predict the kidney area per image slice and kidney span per image volume. They validated the model on a dataset of 90 kidney samples, obtaining a volume estimation accuracy higher than other existing methods. In 2018, Norouzi et al. [27] proposed a fuzzy logic-based model for predicting the renal failure timeframe of chronic kidney disease based on real clinical data, analyzing GFR values. Despite numerous uncertainties related to the variability of the disease course, the model was able to accurately predict changes in GFR over long future periods. Recent and noteworthy applications also pertain to hemodialysis. For example, in 2018, Niel et al. [28] used AI to improve the accuracy of dry weight assessment in hemodialysis patients. They showed that an NN prediction outperformed those of experienced nephrologists in most cases, demonstrating that AI is a powerful predictive tool not only for the onset of kidney disease but also for dialysis treatment.

**Figure 4:**
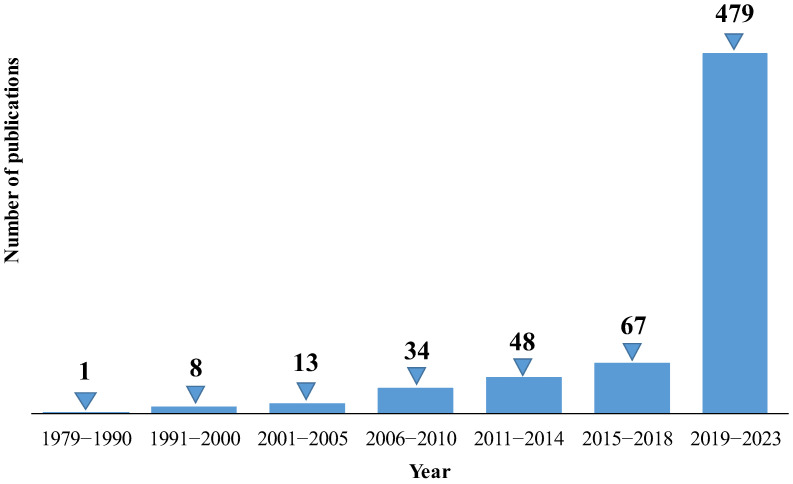
Number of publications per year on Artificial Intelligence (AI) applications related to the topic of kidney disease and relevant therapies in the time interval 1979–2023. The research on the Scopus database used “Artificial Intelligence”, “Kidney Diseases”, and “Hemodialysis” as key search words, limited to English-language publications.

**Figure 5:**
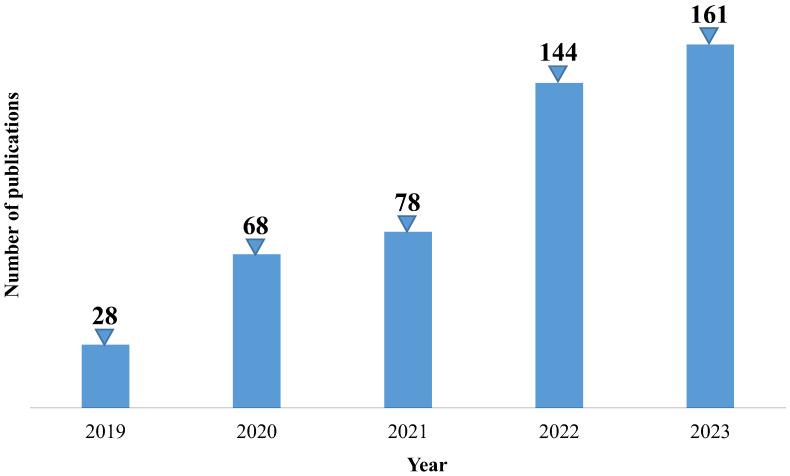
Number of publications per year on Artificial Intelligence (AI) applications related to the topic of kidney disease and relevant therapies in the time interval 2019–2023. The research on the Scopus database used “Artificial Intelligence”, “Kidney Diseases”, and “Hemodialysis” as key search words, limited to English-language publications.

The following figures report the number of publications per year concerning artificial intelligence applications in the renal field in the time intervals between 1979 and 2023 (Figure 4) and, more specifically, 2019–2023 (Figure 5), respectively. The exponential growth of the number of published papers per year highlights the great interest in AI applied to the renal disease and therapy field. Notably, none of the cited publications reported individual-level or anagraphic data, reflecting the authors’ consistent attention to ethical standards aimed at protecting patient privacy. Furthermore, most of the studies included in this paper are based on clinically sourced, validated, and robust datasets, such as “Medical Information Mart for Intensive Care III/IV” (MIMIC-III/IV), and large hospital cohorts, which significantly enhance the reliability and reproducibility of the findings. In cases where smaller datasets were used, their reliability was supported by the reporting of statistical measures such as *p*-values and confidence intervals, strengthening the robustness of the models’ performance.

The breakthrough year seems to be 2022, when the number of published articles increased to 144 (in 2021, it was 78), partly due to the contribution of new transformer variants that have led to superior performance in several applications.

The trend analyzed in the previous histograms confirms the considerable and growing scientific interest in AI themes in this specific field of biomedicine. This vibrant scientific interest justifies the need to review recent literature to capture the current state of the art and identify unresolved aspects that can guide future research endeavors.

The aim of the present review is to analyze the main literature works regarding applications of artificial intelligence in the renal area, considering the most recent time span from 2019 to 2023.

This review is structured in two sections, with a brief concluding discussion on recent developments and emerging trends in artificial intelligence for nephrology, as well as the limitations and challenges of its clinical adoption. The first section offers a concise overview of the primary AI literature models used in the literature and outlines the definition of the associated parameters used for assessing the model’s effectiveness. The second presents the most significant AI literature papers in the renal field, classified as follows:

- AI techniques used as tools for predicting chronic kidney disease in renal-healthy patients or the probability of survival in renal-ill patients, starting from both numeric data and diagnostic images.
- AI techniques used as tools to support traditional methods for the CKD detection in renal-ill patients, considering both numeric data and diagnostic images.
- AI techniques used as tools to improve dialysis treatment or support physicians in managing patients in their care.
- AI techniques used as tools to predict dialysis complications during the treatment or mortality in patients awaiting renal transplantation.

Overall, as will be shown in Section 3, the literature shows a strong focus on the first two categories, with the majority of studies addressing early prediction and diagnosis of kidney diseases. In contrast, research on therapy optimization and complication prevention remains relatively limited, despite its clinical importance, highlighting the need for further studies to develop and validate artificial intelligence tools that can effectively support therapeutic decision-making.

## 2. Machine Learning: Models’ Classification and Performance Parameters

### 2.1. Machine Learning and Its Ramifications

In the renal field, various artificial intelligence models are used for prevention, diagnosis, and treatment. Among these, ML encompasses different types of “learning” approaches [29], each defined by the nature of the training data and the problem addressed. These include supervised, unsupervised, semi-supervised, and reinforcement learning. A separate Section 2.2 is dedicated to **Deep Learning**.

**Figure 6:**
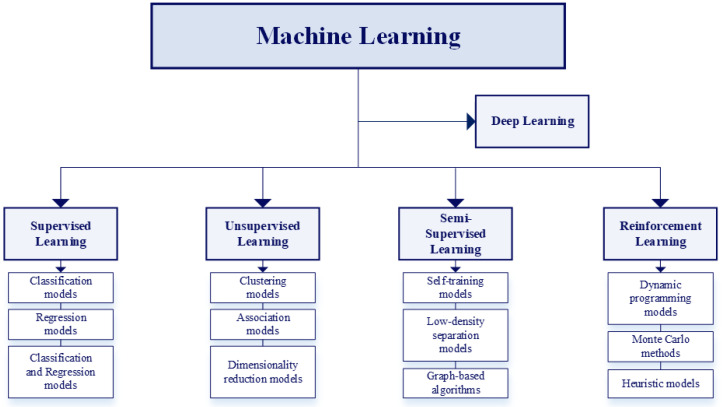
Machine Learning (ML) classification in subcategories, along with the relevant models for each of them.

Figure 6 classifies the different types of ML and the relevant models associated with each category.

- Supervised Learning uses training data that include the desired outputs. It comprises two main types of models: classification, which assigns inputs to predefined categories, and regression, which predicts continuous numerical values based on input features.
- Unsupervised Learning uses training data that do not include the desired outputs. It embraces clustering (based on grouping similar data points), association models (based on identifying relationships between variables), and dimensionality reduction (based on simplifying datasets by reducing the number of features to reduce the computational load) [30].
- Semi-Supervised Learning uses training data that include a small amount of desired outputs. It includes self-training (based on generating new labeled data using self-generated predictions), low-density separation (used in classification problems where classes are separated by low-density regions), and graph-based algorithms (based on the use of graphical representations to model relationships between data instances) [29].
- Reinforcement Learning trains an agent to make decisions in a complex environment. Input data is not provided; instead, only the output is presented. The algorithm learns how to derive this output through trial and error, relying on past experiences. It includes dynamic programming (based on solving problems by breaking them into sub-problems), Monte Carlo methods (based on estimating solutions via random sampling), and heuristic methods (based on approximating solutions when optimal ones are hard to find).

In turn, deep learning includes supervised, unsupervised, and hybrid NNs, which combine models of the previous two classes. In the following section, the supervised learning models are introduced and briefly presented.

#### Supervised Learning Models

**Figure 7:**
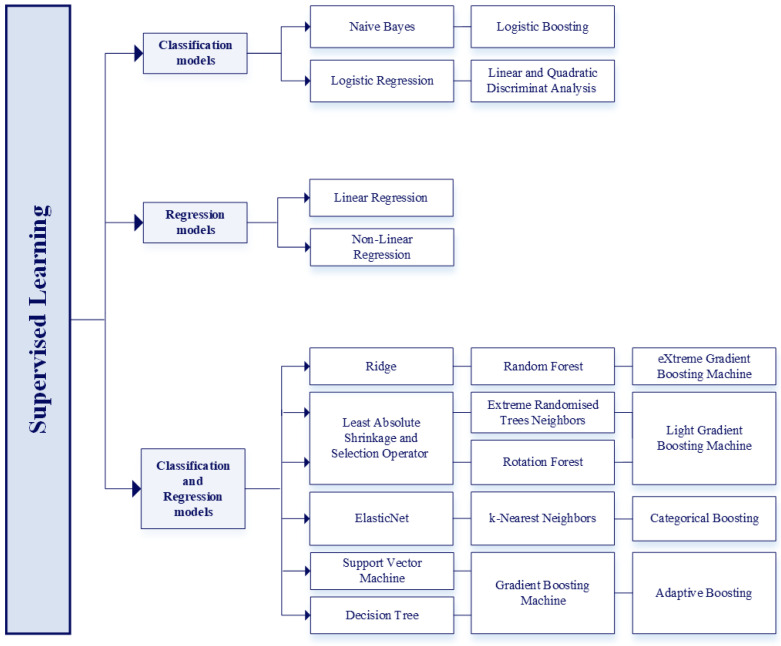
Supervised Learning classification in subcategories along with the relevant models.

Most applications of ML models in the renal field involve supervised learning. Figure 7 reports the most commonly used supervised learning [31] models.

**Classification models**

Naive Bayes (NB) [32] is a simple algorithm based on the assumption of conditional independence between features and on Bayes’ theorem [33]. NB is mainly used for text classification. When applied to data with a normal distribution, it is called “Gaussian”.

Logistic Regression (Log. Reg.) [34] is a linear ML algorithm used for binary classification, predicting the probability that an observation belongs to class 0 or 1. When multiple independent variables influence the outcome, it is called “multivariate logistic regression”.

Logistic Boosting (LogitBoost) [35] improves the performance of weak learning models (often simple decision stumps) by iteratively combining them into a stronger model, reducing classification error.

Linear Discriminant Analysis (LDA) and Quadratic Discriminant Analysis (QDA) [36] are statistical techniques for classification and size reduction, by means of a linear (LDA) or a quadratic (QDA) combination of features separating two or more classes of data, respectively.

**Regression models**

Linear Regression (LR) [37] is a linear model used to predict a continuous outcome (between 0 and 1). Unlike logistic regression, its output spans a range of real values. When multiple independent variables are involved, it is called “multiple linear regression”.

Non-Linear Regression uses powers of the independent variable to model non-linear relationships with the dependent variable. When multiple independent variables are involved, it is called “multiple non-linear regression”. A common form is the “polynomial regression”, which uses a polynomial function to fit the data of one or more independent variables.

**Classification and Regression models**

Ridge [34] is a linear model that incorporates a penalty on the sum of the squares of the model coefficients to reduce overfitting by limiting the model complexity.

Least Absolute Shrinkage and Selection Operator (LASSO) incorporates a penalty on the absolute sum of the coefficients.

ElasticNet combines the penalties of Ridge and LASSO, balancing their strengths to improve generalization in models with many correlated predictors.

Support Vector Machine (SVM) model [38] finds an optimal hyperplane to separate classes (classification) or fit a function (regression), minimizing the prediction error and maintaining an optimal margin.

Decision Trees (DTs) [39] structure decisions in a tree format, where nodes represent a decision based on input features. A type of DT is the Classification And Regression Tree (CART), which constructs binary trees where each node always divides into two branches. Another variant of DT is the Conditional Inference Tree (CIT), which uses statistical tests to choose subdivisions, improving interpretability and robustness.

Random Forest (RF) [40] is an ensemble model that combines multiple decision trees using aggregation (bagging). It aggregates results to improve accuracy and reduce overfitting.

Extremely Randomized Trees (Extra Trees) [41] are similar to RF but introduce additional randomness by selecting both the splitting feature and threshold randomly at each node, increasing diversity among trees.

Rotation Forest [42] is another ensemble method that emphasizes the diversity of trees through data rotation, allowing each base classifier to view the data from a different perspective, improving overall accuracy.

k-Nearest Neighbors (kNN) is an algorithm based on the concept of closeness, assuming that similar inputs yield similar outputs. It uses distance metrics such as Euclidean or Manhattan.

Gradient Boosting Machine (GBM) [40] builds decision trees sequentially, with each new tree correcting the errors of the previous one. In this case, GBM is called “Gradient Boosting Decision Tree” (GBDT).

eXtreme Gradient Boosting (XGBoost) [43] extends GBM with additional features such as tree pruning, parallelization, and Ridge and LASSO regularization, improving robustness and overfitting.

Light Gradient Boosting Machine (LGBM) selects in each tree the leaf with the best result, leading only this one to successive divisions. This approach makes LGBM less conservative but more efficient than GBM.

Categorical Boosting (CatBoost) is optimized for categorical data, automatically handling encoding while using oblique trees (non-orthogonal decision boundaries) to separate different data classes. This minimizes overfitting and training time.

**Figure 8:**
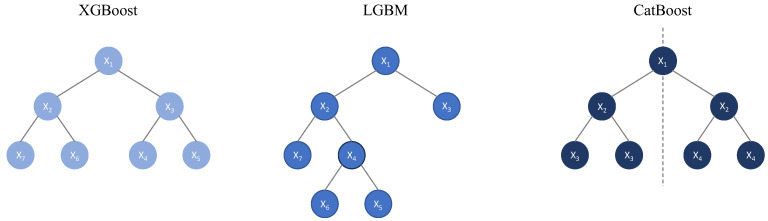
Schematic illustrations of eXtreme Gradient Boosting (XGBoost), Light Gradient Boosting Machine (LGBM), and Categorical Boosting (CatBoost) models. XGBoost and GBM build the trees sequentially and maintain constant tree depth between branches. LGBM creates asymmetrical trees, leading only the best leaf to successive divisions. CatBoost is based on oblique trees, where at a given depth, the same division is used in all branches, as indicated by constant division indices.

Figure 8 shows the different concepts of “tree growth” for the GBM, XGBoost, LGBM, and CatBoost models. The same schematic representation is used for the XGBoost and GBM models, as they are structurally the same and differ only in the calculation mechanisms used.

Adaptive Boosting (AdaBoost) model [40] is similar to the LogitBoost model but provides pure classifications rather than probabilistic estimates for decisions, and is also rarely used as a regressor.

### 2.2. Deep Learning Models

Deep Learning is a sub-class of Machine Learning. It is based on the use of deep ANNs.

**Figure 9:**
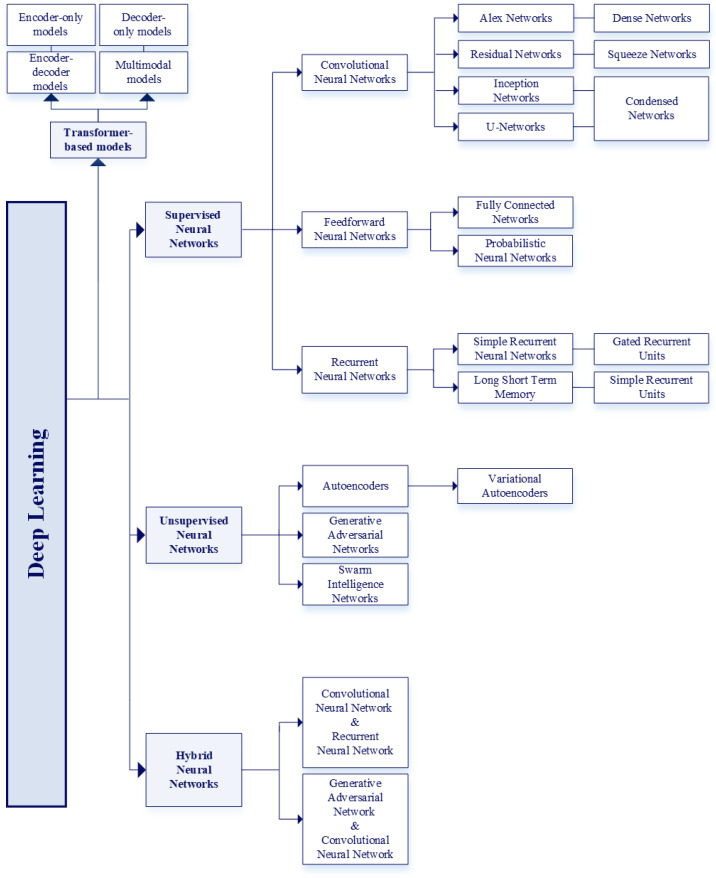
Deep Learning (DL) subcategories and the related models.

Figure 9 reports the classification of deep learning models [44] into supervised, unsupervised, and hybrid neural networks categories, together with the most employed models for each category.

A separate category is dedicated to Transformer-based models, first introduced in 2017 by Vaswani et al. [45]. These models are characterized by the multi-headed attention mechanism, which allows them to focus on multiple and variable-length parts of the input simultaneously. This property has made transformers the foundation for modern Large Language Models (LLMs) such as “Chat Generative Pre-trained Transformer”, best known as ChatGPT.

Transformer-based models can be divided into four main classes [46].

**Encoder-only models** are designed mainly for understanding tasks such as text classification, sentiment analysis, and named entity recognition. They take input sequences, encode them into contextual representations, and output embeddings that capture semantic meaning.

**Decoder-only models** generate text in an autoregressive manner, predicting one token at a time based on the previous context. They are particularly suited for text generation, dialogue systems, and LLM-based applications like ChatGPT.

**Encoder-decoder models** encode an input sequence into a fixed representation and then decode it into an output sequence. They are widely used for machine translation, text summarization, and question answering, and are also called Sequence-To-Sequence (Seq2Seq) transformers.

**Multimodal models** are capable of processing and integrating information from multiple modalities, such as text, images, or audio. They are used in applications like text-to-image generation and vision-language alignment.

These subclasses form the backbone of modern AI systems, enabling highly accurate, flexible, and scalable solutions across NLP, vision, and even multimodal reasoning tasks.

#### 2.2.1. Supervised Neural Networks

Supervised NN models include a variety of architectures suited to different data types and tasks.

**Convolutional Neural Networks (CNNs)** [47] are optimized for gridded data such as images and videos, using convolution layers to detect local patterns (e.g., edges, textures, low-level features). CNNs include the following:

- Alex Networks (AlexNets) were developed by Krizhevsky et al. [48] in 2012. It popularized deep learning in image classification with five convolutional layers.
- Residual Networks (ResNets) use residual blocks to address the gradient degradation, simplifying the back-propagation process during training.
- Inception Networks (InceptionNets), also called GoogLe Networks (GoogLeNets), have multiple convolution modules to capture features at different spatial scales and learn richer representations.
- U-Networks (U-Nets), mainly used for biomedical image segmentation, use a contraction path followed by an expansion path (encoder-decoder), resulting in a typical “U-shape”.
- Dense Networks (DenseNets) connect each convolutional layer to all previous ones within the same dense block, improving learning efficiency.
- Squeeze Networks (SqueezeNets) are similar to AlexNets but smaller in size, used for applications that require low memory usage and high processing speed while maintaining good image classification accuracy.
- Condensed Networks (CondenseNets) reduce parameters and operations through a condensation technique, maintaining high image classification performance with low memory and computational cost.

In **Feedforward Neural Networks (FNNs) [49]**, information moves in only one direction, from input through one or more hidden layers to output. FNNs include:

- Fully Connected Networks, where each neuron in one layer is connected to all neurons in the next layer. A simple form is the MultiLayer Perceptron (MLP), with three layers of nodes: an input layer, one or more hidden layers, and an output layer. MLP is effective for solving general supervised learning tasks.
- Probabilistic Neural Networks (PNNs) provide output probability estimates instead of single predictions. They help to better understand the uncertainty in the data and improve the accuracy of predictions.

**Recurrent Neural Networks (RNNs)** [50] handle sequential or temporal data. The presence of cyclic connections that can maintain an internal memory allows sequences of data to be processed more efficiently. The “bidirectional” variant improves context understanding by processing both forward and backward sequences. RNNs comprise the following:

- Simple Recurrent Neural Networks (SimpleRNNs) are the simplest form of RNNs, where the output of a hidden layer is fed back as input for the hidden layer itself at each time step.
- Long Short Term Memory (LSTM) networks handle long-term time dependencies, suitable for complex sequences. They can also be used in unsupervised scenarios.
- Gated Recurrent Units (GRUs), similar to LSTM, efficiently address the vanishing gradient problem, consisting of the excessive shrinkage of gradients during back-propagation.
- Simple Recurrent Units (SRUs) are faster and less computationally expensive than GRU, ideal for real-time use. They present an internal state that updates efficiently at each time step, allowing relevant information to be stored over time.

#### 2.2.2. Unsupervised Neural Networks

Unsupervised NNs comprise three different types:

**Autoencoders** [44] are composed of an encoder—decoder pair that compresses the input into a more compact representation and then reconstructs it. Different autoencoders are the “Variational AutoEncoders” (VAEs), i.e., neural networks designed to generate new data similar to the training set (e.g., images).

**Generative Adversarial Networks (GANs)** have a generator, which creates realistic data, and a discriminator, which improves its ability to distinguish between real and generated data.

**Swarm Intelligence Networks** [51] are inspired by the behavior of social organisms interacting in a distributed manner to achieve shared objectives. They emulate the way such organisms coordinate their actions to achieve efficient global outcomes without centralized guidance.

#### 2.2.3. Hybrid Neural Networks

Hybrid Neural Networks combine supervised and unsupervised models to handle complex tasks such as image recognition, pattern analysis, and decision making, where traditional neural networks may be insufficient [52]. Two examples of hybrid NNs are as follows:

**CNN-RNN hybrids** [53] combine CNNs (excellent for spatial data like images) with RNNs (designed for sequential data like text), making them suitable when spatial patterns over time must be captured (e.g., videos or audio analysis).

**GAN-CNN hybrids** [54] implement the generator-discriminator structure typical of GANs, where both the generator and discriminator are designed as CNNs. This combination is powerful in image and video generation.

### 2.3. Machine Learning Models’ Performance Indicators

In order to evaluate the efficacy of a model (especially its ability to generalize and perform well on unseen data), various performance indicators are commonly adopted, depending on the type of problem (e.g., classification, regression).

#### 2.3.1. Classification Performance Indicators

Several metrics are used to evaluate the performance of classification ML models [55]. These metrics are typically based on confusion matrix components. In a classification process, positive (i.e., 1) and negative (i.e., 0) refer to the two possible classes. It follows that, in a classification model, a True Positive (TP) is a value correctly predicted as 1, and a True Negative (TN) is a value correctly predicted as 0. On the other hand, a False Negative (FN) is a value incorrectly predicted as 0, and a False Positive (FP) is a value incorrectly predicted as 1. In binary classification, “positive” often indicates the presence of a condition (e.g., disease) and “negative” its absence. In multi-class classification, the concepts generalize to account for multiple classes, allowing for the assessment of classifier performance across different categories without changing their fundamental definitions. Each metric retains its original meaning but is applied to evaluate each class against all others.

**Figure 10:**
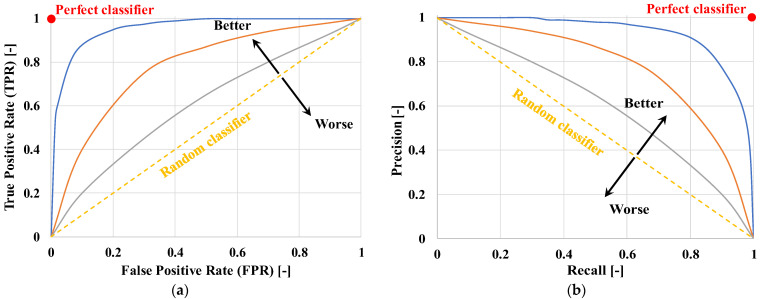
Representation of the Area Under the Receiver Operating Characteristic Curve (AUC-ROC) graph (**a**) and the Area Under the Precision-Recall Curve (AUC-PR) graph (**b**).

- Accuracy is the percentage of correct predictions over total predictions.
- Specificity is the percentage of negative instances correctly identified.
- Recall (or Sensitivity or True Positive Rate (TPR)) is the percentage of positives correctly identified.
- Precision is the percentage of positive instances correctly identified among all predicted positives.
- F_β_-Score combines precision and recall into a single value weighted by β, thus providing an overall measure of the performance. In Equation (6), for β > 1 the score favors recall, while for β < 1 it gives more weight to precision. The most common is the F_1_-Score (β = 1), which gives equal weight to precision and recall.
- False Positive Rate (FPR) is the ratio of the number of cases misclassified as positive to the total number of true negatives.
- Area Under the Receiver Operating Characteristic Curve (AUC-ROC) is the area subtended by the TPR vs. FPR curve, as shown in Figure 10a.
- Area Under the Precision-Recall Curve (AUC-PR) is the area subtended by the Precision vs. Recall curve, as shown in Figure 10b.

- Average Precision (AP) is the precision averaged across different Recall levels along the Precision-Recall curve.
- The mean Average Precision (mAP) is the average of AP across all classes, used to assess the performance of classification or object detection models.
- The Dice coefficient is used in image analysis; it assesses how closely a segmented region of an image coincides with a reference or truth region.
- Matthews Correlation Coefficient (MCC) measures the correlation between the predictions of a binary classification model and the actual class labels, returning a value from −1 (inverse prediction) to +1 (perfect prediction), with 0 indicating a random prediction.

The mathematical expressions to calculate the classification ML models’ performance indicators are reported in Table 1.

#### 2.3.2. Regression Performance Indicators

To evaluate the performance of a regression ML model, several standard metrics are commonly used [55]:

- R^2^ Score measures how well a statistical model predicts an outcome. R^2^ ranges between 0 and 1: if R^2^ = 0, the model does not predict the outcome; if R^2^ = 1, the model perfectly predicts the outcome; if 0 < R^2^ < 1, the model does not predict the outcome perfectly.
- Mean Squared Error (MSE) calculates the mean square of the differences between predicted and actual values.
- Mean Absolute Error (MAE) averages the absolute differences between predicted and actual values.
- Root Mean Squared Error (RMSE) measures the square root of MSE between predicted values and actual values.
- Concordance index (C-index) measures how well a model predicts the order of events (e.g., survival time of patients). It compares the model predictions with what actually happens. It is the ratio of concordant pairs between real and predicted labels to the total number of comparable pairs.

The mathematical expressions to calculate the ML regression models’ performance indicators are reported in Table 2.

### 2.4. Comparative Insights: The Main Machine Learning Models Applied to Nephrology

Although a wide range of machine learning models and performance metrics is available, the nephrology literature consistently shows a preference for a limited set of models that are repeatedly applied across different studies. This preference is guided by factors such as the nature and size of available datasets, the need for model interpretability to support clinical decision-making, and the trade-off between predictive performance and implementation feasibility [56,57].

As will be shown in detail in Section 3, supervised learning dominates nephrology applications. Specifically, logistic regression and regularized approaches such as LASSO, Ridge, and ElasticNet are highly used because they provide coefficients that are easy to interpret, perform robustly even with small or moderately imbalanced datasets, and are already familiar to clinicians [57,58]. However, their main limitation is the inability to fully capture complex nonlinear relationships between variables.

Tree-based methods, including decision trees, random forests, and boosting algorithms, such as XGBoost, LightGBM, and CatBoost, are now among the most widely used approaches for structured Electronic Health Record (EHR) and laboratory data. These methods handle missing values efficiently, are accurate and robust to outliers, moderately interpretable, and provide feature importance measures that can highlight the most relevant clinical predictors [56,59], but may overfit if not well-tuned and cross-validated [57].

When larger datasets are available, neural network-based models are increasingly explored. Feedforward artificial neural networks (ANN, MLP) can capture complex non-linear patterns in clinical data and have shown very high accuracy in chronic kidney disease staging and prognosis prediction, although they remain less interpretable and more sensitive to hyperparameter choices than tree-based models [57].

For imaging tasks, deep learning has become the dominant paradigm. CNNs and their derivatives (U-Net, ResNet, DenseNet) automatically extract hierarchical features from biopsy slides, CT scans, and Magnetic Resonance Images (MRIs), achieving excellent segmentation and classification performance. More recently, transformer-based architectures, such as Vision Transformers (ViTs) and multimodal attention-based models, are being explored for Whole-Slide Image (WSI) classification and for integrating imaging with clinical features, obtaining promising results [60]. However, these approaches require large, well-annotated datasets and substantial computational resources, and their “black-box” nature can limit trust among clinicians [56].

To address this limitation, recent years have seen a growing adoption of eXplainable AI (XAI) techniques, such as SHapley Additive exPlanations (SHAP), Local Interpretable Model-agnostic Explanations (LIME), Gradient-weighted Class Activation Mapping (Grad-CAM), and attention-based mechanisms, which aim to make model decisions more transparent and interpretable, and help clinicians understand which features or image regions drive predictions [61,62,63].

Sequential and temporal models, such as SimpleRNN, LSTM networks, and GRU, are particularly valuable for modeling longitudinal data streams, including dialysis session records or Intensive Care Unit (ICU) monitoring data. These models capture time dependencies better than static approaches, but they are computationally demanding, hardly interpretable, and more prone to overfitting if data are scarce [60].

Finally, although they have lower transparency, hybrid models are emerging as promising and accurate strategies for integrating multimodal information (demographic, laboratory, imaging) into a single predictive pipeline and for quantifying predictive uncertainty, which is crucial for clinical decision support [56].

As a summary, Table 3 provides a comparative overview of the model families and parameters presented in Section 2.2 and Section 2.3, most frequently applied in nephrology, summarizing their strengths, limitations, typical applications, and metrics used to evaluate their performance.

## 3. Machine Learning Models Used as Prediction, Detection, and Treatment Support Tools in the Renal Field

Chronic kidney diseases affect about 13.50% of the world’s population [64] at different severity stages, causing millions of victims each year and health care costs of about 140 billion euros just in Europe alone [65]. However, their progression can be slowed, and complications minimized if detected early. Therefore, nephrologists aim to decrease the mortality rate of patients by implementing preventive protocols and closely monitoring their condition.

Acute kidney injuries affect about 15% of all hospitalizations, up to 50% of patients admitted to the ICU [66], and can increase the risk of mortality in patients with end-stage renal disease or chronic kidney disease who require dialysis or kidney replacement.

Therefore, artificial intelligence—and, more specifically, machine learning algorithms—represents an efficient tool to predict early CKD, AKI, and their progression.

Predictions can be based on numerical data or diagnostic images. In the first case, starting from data such as patients’ cardiovascular conditions and glomerular filtration rate measurements, the output typically entails predicting an imminent abnormal condition for the patient, whether it is the onset of a disease or the degeneration of an already compromised state. In the second case, using CTs or MRIs of the kidneys, supervised DL models can be used for pre-classification of kidney allograft biopsies, as models to predict renal survival after transplantation, for scanning all renal biopsies, or for applying certain basic algorithms before examination by the pathologist.

The follow paragraphs focus on the main applications in the field of chronic kidney disease and acute kidney injury as an aid to the individual physician’s prediction of the future renal status of healthy or CKD patients (Section 3.1), the detection of chronic kidney disease in renal-ill patients in support of traditional methods (Section 3.2), the identification of the most suitable treatment options for them (Section 3.3), and the analysis of possible complications related to therapy (Section 3.4). At the end of each section, up to Section 3.4, a summary table will summarize the papers mentioned in that paragraph. Finally, a discussion has been conducted on recent developments in AI (Section 3.5) and on the main limitations and challenges related to the adoption of AI in clinical practice (Section 3.6).

### 3.1. Machine Learning as a Tool to Predict Kidney Diseases

ML models have been applied to the prediction and classification of hospital data to identify problems such as cases of chronic kidney disease [67,68,69] or, more specifically, kidney cancers [70,71,72,73], also through the use of innovative approaches. High performances were obtained in many of these studies, as shown by an AUC-ROC value of 98.86% using a Deep Neural Network (DNN) [68], F_1_-Score values greater than 96% using a convolutional neural network [69], or else accuracy values greater than 95% using complex approaches as transfer learning [72] or D-ACO [67]. In detail, D-ACO is a hybrid ML algorithm composed of the Density-based Feature Selection (DFS) algorithm and Ant Colony-based Optimization (ACO), algorithms used especially in the medical field for feature selection and classification, respectively, while transfer learning is a deep learning approach that uses pre-trained models as a starting point for training algorithms with similar and related goals, so as to reduce the time and data needed for training. In this regard, for the implementation of the final model, Nasir et al. [72] used an AlexNet model combined with Stochastic Gradient Descent with Momentum (SGDM), ADAptive Moment estimation (ADAM), and Root Mean Square PROPagation (RMSPROP), which are optimization algorithms able to improve training efficiency by stabilizing convergence and dynamically adjusting learning rates through the strategic use of cost function gradients.

Lower, but still good efficiency parameters were obtained in studies [70,71], such as an accuracy of 83.43% using a MultiModal Deep Learning Model (MMDLM) composed by a ResNet and a fully connected network, an AUC-ROC of 65.10% using a clustering algorithm combined with a LASSO regressor, or a Dice score of 87% using a “V Bottleneck Multi-resolution and Focus-organ Network” (VB-MrFo-Net) demonstrate. Specifically, a VB-MrFo-Net is a model implemented by Chen et al. [71] to segment specific regions of the affected organs thanks to its “bottleneck” function, which reduces data dimensionality while preserving the most important information to optimize the accuracy of the analysis. After the segmentation, the combination of an RF model and an SVM model allowed them to predict the clear-cell Renal-Cell Carcinoma (ccRCC) prognosis and its stage with an AUC-ROC value of 78.20%.

In the context of AI, a trained model can be defined as validated when it is subjected to clinical tests, which, to our knowledge, have not yet been addressed in these studies. This aspect becomes even more important when data from a single database is used for training, as it is potentially responsible for the high-performance efficiencies of the models, as shown in the studies [67,68,69,72]. On the contrary, the presence of data from several hospitals reduced the quality of the models [70,71] in the training phase, but potentially contributed to the implementation of models with a higher capacity for generalization. To prove this, it would be necessary to subject the analyzed studies to clinical trials.

**Figure 11:**
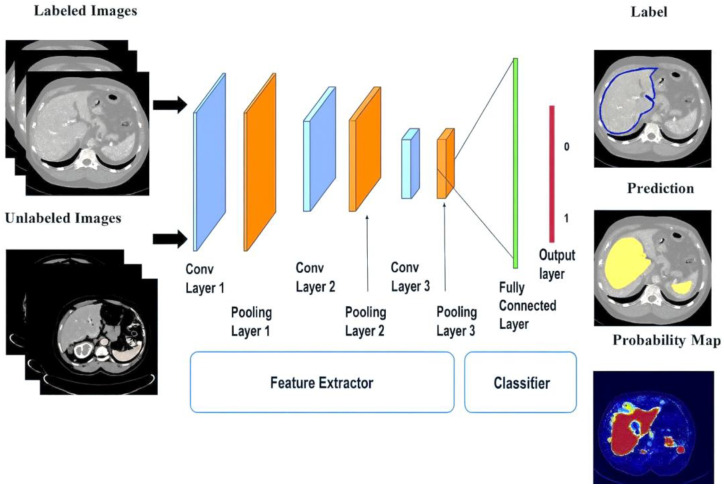
Example of a Convolutional Neural Network (CNN) as a tool for the prediction of kidney cancer, identifying the interested region through the so-called “automatic segmentation” applied on Computational Tomography (CT) images. Reproduced from Chen et al. [73].

In conclusion of this first block of works, a last interesting study was conducted by Chen et al. [73], who used the so-called “Adaptive Hybridized Deep Convolutional Neural Network” (AHDCNN) on CT images to predict kidney cancer, as shown in Figure 11.

Specifically, AHDCNN is a convolutional neural network with three stages composed of pooling layers and linear convolutions. used in this study to reduce the size of the images and to extract their most important features, respectively. After training, AHDCNN achieved an accuracy of 97.14% in tumor early recognition, thus anticipating the possible achievement of its final stage.

Other authors [74,75,76,77,78,79,80,81,82,83,84,85,86,87] compared several models to identify the best performance in predicting the onset of chronic kidney disease, achieving accuracy [74,77,79,82,84,85] and F_1_-Score values [75,76,86,87] over 96% using deep neural networks [76,79], or simpler machine learning models [74,77,78,81,84,85,86,87], all of which are shown in Table 4 with the respective authors.

Among these studies, particularly relevant was the research conducted by Islam et al. [75], who implemented a classification and rule generation analysis on a CKD dataset. Their objective was to test different models for the accurate and early identification of chronic kidney disease cases on new data and to derive decision rules illustrating relationships between CKD attributes. For classification, they compared two boosting algorithms, AdaBoost and LogitBoost, while for rule generation, they evaluated two rule induction methods, J48 and Ant-Miner. The dataset consisted of records from 2800 patients, each with 24 attributes. The LogitBoost and Ant-Miner combination achieved the highest performance, with a predictive F_1_-Score of 99.75%, and identified nine decision-making conditions based on patient attributes as the most relevant for CKD prediction.

With the same aim, Bandera et al. [88] and Pati et al. [89] implemented ML models combined, respectively, with pre- and post-processing algorithms able to further improve the performance of the predictive models, as accuracy and F_1_-Score values higher than 98.50% demonstrate. Specifically, Bandera et al. [88] used an AdaBoost model in combination with “Decision Making Trial And Evaluation Laboratory” (DEMATEL) neutrosophic, an algorithm capable of selecting the most relevant features, while Pati et al. [89] combined an ANN with Voting and Bagging techniques, ensemble methods of equal (Bagging) or different models (Voting) used to improve the prediction accuracy achieved by the ANN model.

In a similar way, Poonia et al. [80] and Rashid et al. [82] developed a model for the prediction of chronic kidney disease by combining an artificial neural network with Chi-square and Particle Swarm Optimization (PSO), respectively, which are algorithms able to select the most relevant features in the pre-processing stage prior to model application. Accuracy values up to 99.76% were obtained.

Priya et al. [83] also developed a model for predicting and classifying chronic kidney disease, but combining an ANN model with a “Hybrid Gravitational Search Algorithm and Particle Swarm Optimization” (HGSAPSO), an algorithm composed of the Gravitational Search Algorithm (GSA), which was used to explore the solution space to identify optimal solutions for the classifiers’ parameters, and the PSO, which was capable of refining the search for solutions by focusing on the most promising ones. Despite the development of such a model supported by optimization algorithms, this case yielded inferior results compared to what had been seen previously [80,82], with an accuracy of 93%. This is likely due to the lack of a pre-processing algorithm to select the most relevant features prior to model application, a crucial step for an ANN to mitigate the effects of noise, struggling to identify patterns and relationships in the data.

Following this topic, Lakshmanaprabu et al. [90] proposed in 2019 a model able to predict not only the onset of a kidney disease, but also its level of severity using the Internet of Things (IoT), i.e., devices used to collect real-time health data from patients, and cloud systems, used for their storage and processing. Specifically, the implementation of a deep neural network combined with the PSO algorithm, implemented to select the most relevant features on which to apply the model, allowed the researchers to obtain an accuracy value of 99.25%.

Although the results and the ambitious aims, many of the studies described here have some common limitations, which have been partially discussed above, as the presence of data from a single database, which reduces the generalizability of the results, and the absence of clinical studies that can validate the models in real-world settings.

Another critical issue is the poor interpretability of some models, particularly deep learning models [76,79], and the disequilibrium in all datasets used, characterized by more data from CKD patients than from unaffected patients, an aspect that can distort model performance.

In addition, the lack of cross-validation, a key technique for assessing model robustness by splitting the dataset into multiple subsets, prevents reliable performance estimates in some models [76,77,78,79,80,82,83,84,85]. Without this practice, it is more difficult to ensure that the results are representative. Finally, the limited variety of features considered in all these studies may lead to an incomplete understanding of the problem. Addressing these limitations is crucial to improving the reliability and applicability of the analyzed models, which, as shown by the excellent results obtained, possess highly efficient potential.

Despite the presence of some of the limitations already described, the studies conducted by Hamedan et al. [91] and Arulanthu et al. [92] also demonstrated a good validity of the implemented systems and a good predictive ability, with accuracy values of up to 97.75%, using a simple fuzzy system model [91] and a logistic regression model [92], respectively.

Due to the increase in kidney disease cases [13] over the years, the need to develop predictive models for healthy people has become increasingly relevant. Thus, some models were developed capable of identifying in healthy individuals those most prone to the development of CKD [93,94] or AKI [95]. Specifically, a C-index value of 80% was obtained by Bell et al. [95] using a multivariate logistic regression model, while the use of more complex convolutional neural networks allowed Marechal et al. [94] to obtain an AUC-ROC value of 92%. Also innovative was the approach used by Kanda et al. [93], who implemented a Bayesian neural network to assess causal relationships between input and output variables, followed by an SVM to predict chronic kidney disease progression, resulting in predictive errors close to zero.

Furthermore, Bermudez-Lop et al. [96] demonstrated that additional parameters, such as lipoprotein composition and the number of lipid particles in this case, have a greater discrimination capacity with respect to traditional parameters alone. Specifically, in a sample of 209 patients with chronic kidney disease and 186 healthy controls, all non-diabetic, participants’ blood lipids were analyzed with Nuclear Magnetic Resonance (NMR) spectroscopy, an analytical technique used to determine the molecular structure of chemical compounds. The data obtained from these analyses were used in an RF model to assess the ability to distinguish patients with chronic kidney disease from healthy individuals, showing that blood lipid content was a good discriminator for this classification.

In recent years, many researchers have also used ML models to predict CKD or AKI in patients already suffering from other diseases or to study the link between renal diseases and the onset of seemingly unrelated diseases.

For AKIs, some authors developed models to predict acute kidney injury in people who have sustained burns [97], in sepsis survivors [98], or in patients undergoing surgery [99,100,101], with a focus on patients undergoing liver transplantation [102,103,104] or hypotensive patients [105]. Among these studies, an AUC-ROC value of 92% was achieved by Rashidi et al. using a deep neural network [97], followed by lower AUC-ROC values (of up to 86%) obtained with simpler models as random forest [102], eXtreme gradient boosting [98], artificial neural network [104], gradient boosting machine [99,103], and the so-called “Intraoperative and Data Embedded Analytics” (IDEA) [100]. Specifically, IDEA is a simple but innovative model implemented by Adhikari et al. [100] combining random forest with a Generalised Additive Model (GAM), a statistical model capable of modeling linear and non-linear relationships between inputs and outputs. In this study, GAM was used to study the relationships between the pre-operative data of the patients analyzed and the risk of developing acute kidney injury. After that, random forest allowed the pre-operative risk calculated by GAM to be combined with physiological variables collected during the operation, updating the prediction of AKI risk and obtaining an AUC-ROC of 86%.

A strong interest has also been reserved for predicting renal survival after kidney transplantation [106,107] and renal aneurysm repair [108], or the development of fatal pathologies, such as pneumocystis carinii pneumonia [109] and Cardiac Surgery-Associated Acute Kidney Injury (CSA-AKI) [110,111]. Given the complexity of all these diseases, supporting studies have obtained slightly lower but still useful results than those aimed at predicting non-fatal kidney disease, as evidenced by the AUC-ROC value of 83.90% [110] obtained with a RF model or the accuracy of 82.10% [111] and 86% [108] obtained with a XGBoost model called “Detect-A(K)I” by the authors and an ANN model, respectively.

**Figure 12:**
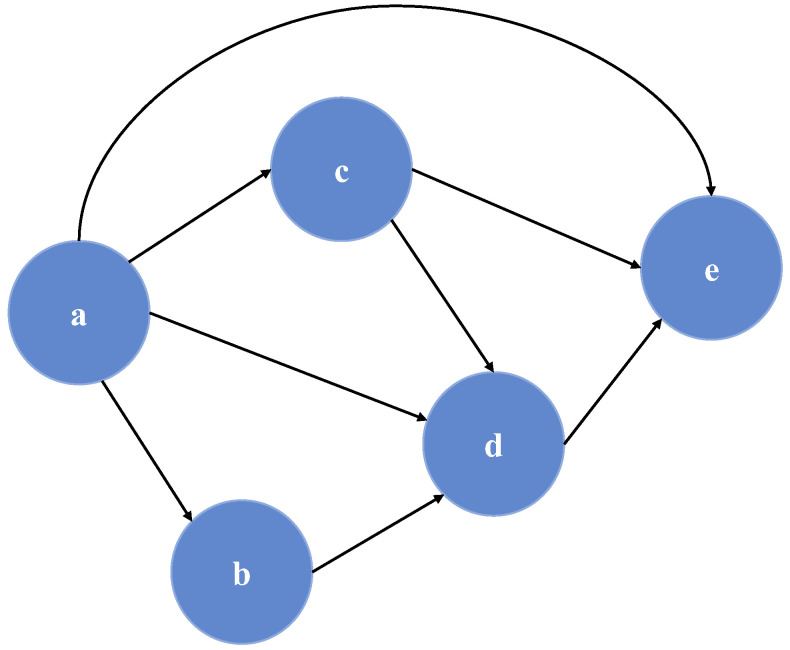
Schematic representation of a joint Bayesian model, where nodes (classified from “a” to “e”) represent random variables and directed edges represent probabilistic dependencies between variables. A directed edge from a node to another indicates that the second node is conditionally dependent on the first one.

Particularly effective was the use of the joint Bayesian model, a statistical model that allows multiple variables to be analyzed together, using probability theory to update knowledge as new data were collected. Figure 12 presents the typical structure of a joint Bayesian model.

Used to predict renal survival of transplant patients [107], this model, renamed by the authors as “Dynamic Integrative System for Predicting Outcome” (DISPO), provided an AUC-ROC value of 85.70%.

Among the mentioned studies, another relevant approach was that used by Paquette et al. [106], who employed innovative neural networks such as DeepHit and DeepSurv to achieve the same predictive survival goal. Specifically, DeepSurv is a neural network that learns how variables influence the risk of an event, whether in a linear or non-linear manner. In contrast, DeepHit is a more flexible neural network that can analyze the probability of an event occurring at various time points, without adhering to strict rules regarding the data. This network obtained a higher C-index of 66.10%, higher than the 65% obtained by the DeepSurv model.

In regard to acute injuries, the link between COVID-19 and the possible occurrence of acute kidney injury in hospitalized patients has recently attracted the attention of many researchers, including Naser et al. [112] and Lu et al. [113], who addressed the problem using a multivariate logistic regression model and the combination of an LSTM model with an FNN model, respectively. After analyzing data from laboratory tests, medical conditions, and demographic information of several patients, Lu et al. [113] obtained a model that was able to predict the occurrence of acute kidney injury in COVID-19 patients with an AUC-ROC of 96.50%, while Naser et al. [112] obtained an odds ratio between AKI patients died and no-AKI patients equal to 48.6, where odds ratio is a statistical measure used to quantify probabilities of a given event between two groups, especially in epidemiology and medicine. The predictability of acute kidney disease events in the first study and the high odds ratio of the second one proved the expected strong correlation between AKI and COVID-19.

In regard to chronic diseases, predictive models of the kidney function deterioration in sepsis survivors [114] or in patients with Immunoglobulin A Nephropathy (IgAN) [115,116], CKD of unknown etiology (CKDu) [117], hyperkalemia (potassium > 5.5 mmol/L) [118], diabetes [119], or kidney cancer [120] have been implemented. Using simpler ML models as gradient boosting decision tree [114], called “Landmark-Boosting” by the authors and designed specifically to handle time data [119], random forest [115], ANN [116], XGBoost [118], or more complex deep neural networks [117,120], the authors obtained excellent results, such as an AUC-ROC of 95.70% [118], or an accuracy close to 90% [117].

Innovatively, the implementation of the DIstillation with NO labels-Vision Transformer (DINO-ViT) model performed by Wessels et al. [120] enabled them to predict survival in renal cell carcinoma patients. In detail, DINO is a self-supervised, label-free model capable of extracting key features from images of interest, used by the authors prior to applying the transformer. The resulting model provided reliable results, such as a hazard ratio of up to 2.31 for the patients involved, where hazard ratio refers to a measure of the hazard rate (for survival, in this case) of a treatment group compared with a healthy control group. The reliability of the model confirmed the good interpretability of the images produced by the model, which could thus be used as a potential tool to support the disease investigation carried out by medical doctors.

On the contrary, some applications have focused on the prediction of pathologies in patients already suffering from chronic kidney disease, such as hyperkalemia [121] or diabetes [122], since diabetes and CKD together are closely associated with increased mortality risk. Accuracy [122] or AUC-ROC [121] values of up to 89.15% were obtained in these studies.

Other studies proposed artificial intelligence models to monitor eGFR [123] and other factors for predicting future renal deterioration in hospitalized [124,125,126,127] and ICU patients [128,129,130,131,132]. Given the variability of the patients involved, highly variable predictive performances were obtained in the studies cited, such as AUC-ROC values ranging from 73% [124] to 96% [132]. Among the various ML models used, such as random forest [124,132], XGBoost [131], multivariate logistic regression [128], or neural networks [126,127,129,130], particularly innovative approaches were used by Lee et al. [123], Song et al. [125], and Kandasamy et al. [127]. Specifically, the use of the probabilistic Gaussian Mixture Model (GMM) clustering model in combination with a hybrid neural network based on the use of radial functions allowed Kandasamy et al. [127] to analyze in 40,000 ICU patients the progression of their CKD condition. On the other hand, the development of Deep Support-Gradient Boosting Trees (DS-GBT), a hybrid model combining deep learning and decision trees, enabled Song et al. [125] to predict the development of acute kidney injury in hospitalized patients 48 h in advance, achieving an AUC-ROC of 95%. Finally, the implementation of an unsupervised machine learning model based on the “Bag-of-Words” (BoW) model helped Lee et al. [123] to predict the change in renal function after 1 year based on relevant visual features in renal biopsy specimens of CKD patients. In this work, BoW identified and grouped image segments into “visual words” to construct a representative visual vocabulary of kidney biopsies, after the application of a pre-trained neural network able to extract visual features from image segments. The application of a clustering algorithm allowed them to group similar visual segments together to find recurrent patterns. The development of an RF model finally predicted renal function at the time of biopsy and after one year in terms of eGFR, with good AUC-ROC values of 93% and 80%, respectively.

During the same years, some authors have also proposed models able to identify risk factors associated with the rapid decrease in renal function among chronic kidney disease patients [133] considering their CKD severity stage [134,135,136], until end-stage kidney disease condition [137] and mortality [138]. AUC-ROC values equal to [133,134,137], or greater than [135,136] 90% were obtained, and several innovative models were proposed. In this regard, Akter et al. [136] proposed CKD.Net, a model capable of predicting the stages of chronic kidney disease using the combination of a SimpleRNN and an MLP. The implementation of this model allowed the authors to achieve an accuracy of 99.80% in predicting the five chronic kidney disease classes in patients aged from 25 to 90. Bellocchio et al. [137] proposed PROGRES-CKD, a Naive Bayes classifier used on a dataset of adult patients. Using 34 features of 24,535 patients, the model was able to predict the onset of end-stage kidney disease within 6 and 24 months with an AUC-ROC of 91% and 85%, respectively.

Further studies have focused on the problem of kidney stones, a very common renal disease that causes the accumulation of small crystals in the kidney due to an inadequate diet or poor urination.

Using images, it was possible to create models to predict post-operative outcomes after the treatment of large kidney stones through the so-called “PerCutaneous NephroLithotomy” (PCNL) [139] or stones composition with a highly specific, non-invasive approach [140]. For these goals, Black et al. [140] used a ResNet model, while Shabaniyan et al. [139] used an SVM model combined with Sequential Forward Selection (SFS) and Fisher Discriminant Analysis (FDA), algorithms capable of reducing the number of features to be considered and increasing the separability of classes in the classification process, respectively.

In both cases, the predictive capacity of the models proved to be particularly good, obtaining an accuracy value of 94.80% and a recall value of 85%, respectively.

Prediction of possible post-transplant renal rejection through AI analysis of kidney allograft biopsies has also attracted great interest. Histopathological evaluation of transplant biopsies is the most widely used method to diagnose allograft rejection, but it requires expertise and time that can be reduced by using artificial intelligence algorithms, as performed by Kers et al. [141]. They implemented a CNN model to predict possible allograft rejection in test patients, obtaining an AUC-ROC value of 78%, which was sufficient to confirm the discrete generalization ability of the model.

Table 4 reports a list of all quoted literature works, providing key details on adopted models, a brief description of the dataset employed and their origin, the number and type of features used to train the model, the results, in terms of performance parameters, obtained by the best performing model among those tested, and, in the last column, the purpose of the work.

**Table 4:** Literature overview on Machine Learning (ML) models developed for kidney disease prediction. Legend: n.a. = not available.

Authors and Ref.	Year of Publication	Models	Dataset	Input Variables	Best Results	Aim
Adhikari et al. [100]	2019	IDEA model	2911 adult surgical patients from the University of Florida Health, FL, USA	285 (pre-operative, and intraoperative variables)	AUC-ROC: 86% Accuracy: 78%	Early prediction of post-operative AKI in patients undergoing surgery
Akter et al. [136]	2023	CKD.Net model	Regular health check information of 1 million CKD patients from several (unspecified) hospitals of the National Health Insurance Sharing Service (NHISS) website, released by Wonju-si, Gangwon-do, Republic of Korea	27 (demographic information, clinical variables, and laboratory data)	Accuracy: 99.80%	Prediction of different CKD stages and monitoring of eGFR and creatinine levels in patients aged 25 to 90
Akter et al. [79]	2021	ANN model LSTM model Bidirectional LSTM model GRU model Bidirectional GRU model MLP model SimpleRNN model	400 records from the ML repository of the University of California, Irvine, CA, USA	25 (demographic information, clinical variables, and laboratory data)	Accuracy (ANN model): 99%	Early diagnosis of CKD and identification of the associated risk factors based on patients’ data
Alfieri et al. [129]	2021	CNN model Log. Reg. model	35,573 ICU patients from the electronic Intensive Care Unit (eICU) database, released by Massachusetts Institute of Technology, Cambridge, MA, USA + MIMIC-III database, released by Beth Israel Deaconess Medical Center (BIDMC), Boston, MA, USA	Demographic information, and laboratory data (n.a. for CNN model, 11 for Log. Reg. model)	AUC-ROC (CNN model): 89%	Prediction of AKI in ICU patients based on changes in urinary flow
Alfieri et al. [130]	2022	Log. Reg. model DL model	10,596 ICU patients from the University Hospital ICU of Amsterdam, Amsterdam, Netherlands	Hourly urine output values, creatinine levels, and demographic data	AUC-ROC (DL model): 90.70%	Prediction of AKI patients based on serum creatinine and decrease in urine output
Almansour et al. [77]	2019	ANN model SVM model	400 records from Apollo Hospitals, Tamil Nadu, India, released by the ML repository of the University of California, Irvine, CA, USA	24 (demographic information, clinical variables, and laboratory data)	Accuracy (ANN model): 99.75%	Early diagnosis of CKD patients to reduce the risk of progression to chronic renal failure
Arulanthu et al. [92]	2020	Log. Reg. model	400 records from the ML repository of the University of California, Irvine, CA, USA	24 (demographic information, clinical variables, and laboratory data)	Accuracy: 97.75%	Prediction of CKD patients via IoT devices and cloud platforms
Arumugham et al. [68]	2023	DNN model	400 records from the ML repository of the University of California, Irvine, CA, USA	25 (demographic information, clinical variables, and laboratory data)	AUC-ROC: 98.86% Accuracy: 98.75%	Prediction of early-stage CKD via explainable models based on patients’ data
Bandera et al. [88]	2023	GBM model AdaBoost model RF model (all preceded by the Neutrosophic DEMATEL algorithm)	400 records from the ML repository of the University of California, Irvine, CA, USA	28 (demographic information, clinical variables, and laboratory data)	Accuracy (Neutrosophic DEMATEL algorithm + AdaBoost model): 99.17%	Prediction of CKD progression considering only the most relevant features of patients’ data
Bell et al. [95]	2020	Multivariate logistic regression model	273,450 adult patients from hospitals in Tayside, Scotland, UK + 218,091 patients of hospitals in Kent, England, UK; and 1,173,607 adult patients from hospitals in Alberta, Canada, both for model validation	4 (demographic information, previous pathologies, and laboratory data)	C-index: 80% (Tayside)	Prediction of the development of AKI based on serum creatinine values of adult patients
Bellocchio et al. [137]	2021	PROGRES-CKD model	24,535 CKD patients from Fresenius Medical Care’s (FMC) NephroCare network in Europe + 6760 patients from German Chronic Kidney Disease (GCKD), Germany	34 (demographic information, previous pathologies, clinical variables, and laboratory data)	AUC-ROC (GCKD): 91% (6 months), 85% (24 months)	Prediction of ESKD in CKD patients at 6 and 24 months
Bermudez-Lop et al. [96]	2019	NMR spectroscopy + RF model	395 non-diabetic individuals from NEFRONA cohort including different hospitals, Spain	17 (demographic information, previous pathologies, clinical variables, and laboratory data)	AUC-ROC: 78.90%	Prediction of the risk of atherosclerosis in non-diabetic CKD patients
Black et al. [140]	2020	ResNet model	127 digital renal images of 63 human kidney stones from the stone laboratory Louis C. Herring and Co., Orlando, FL, USA	Structural and morphological features extracted by the model	Recall: 85%	Prediction of human kidney stones composition from digital renal photographs
Bredt et al. [104]	2022	Log. Reg. model ANN model	145 Deceased-Donor Liver Transplantation (DDLT) cases from a tertiary referral hospital, Brazil	6 (demographic information, clinical variables, and diagnostic data)	AUC-ROC (ANN model): 81%	Prediction of AKI after liver transplantation in transplant patients
Chen et al. [71]	2023	VB-MrFo-Net model + RF model + SVM model	126,345 CT images of 838 patients from cohorts of Shanghai General Hospital, China + The Cancer Genome Atlas (TCGA), USA + Clinical Proteomic Tumor Analysis Consortium (CPTAC), USA + Kidney Tumor Segmentation Challenge, USA	2600 (VB-MrFo-Net model; tumor structure and texture information extracted by the model) 22 (RF model + SVM model; tumor structure and texture information)	Dice score: 87% (VB-MrFo-Net model) AUC-ROC: 78.20% (RF model + SVM model)	Non-invasive segmentation and prediction of ccRCC prognosis and its stage based on patients’ data
Chen et al. [73]	2020	AHDCNN model	CT and MRI images of 100 patients from National Institutes of Health (NIH) Clinical Center, Bethesda, Maryland, MD, USA	Renal nodules structure and texture information extracted by the model	Accuracy: 97.14% F_1_-Score: 97.30%	Early diagnosis of CKD patients, with attention to kidney cancer and its subtypes using IoT platform
Elhoseny et al. [67]	2019	D-ACO model	400 records from the ML repository of the University of California, Irvine, CA, USA	24 (demographic information, clinical variables, and laboratory data)	Accuracy: 95%	Prediction and classification of CKD based on patients’ data
Galloway et al. [121]	2019	CNN model	1,638,546 ECGs of 511,345 patients from three Mayo Clinic centers in MN, FL, and AZ, USA	4 (ECG leads)	AUC-ROC: 88.30%	Prediction of hyperkalemia in patients with renal disease based on potassium level
Hamedan et al. [91]	2020	Fuzzy system model	216 kidney disease patients from two teaching hospitals in Tehran, Iran	16 (demographic information, clinical data, laboratory data, and previous pathologies)	Accuracy: 92.13% AUC-ROC: 92%	Prediction of CKD based on patients’ data
He et al. [102]	2021	RF model SVM model DT model CIT model Log. Reg. model	493 Donations after Cardiac Death Liver Transplantation (DCDLT) patients from the First Affiliated Hospital, Zhejiang University School of Medicine, Hangzhou, Zhejiang, China	51 (demographic information, pre-operative, intraoperative, and post-operative data)	AUC-ROC (RF model): 85% Accuracy (RF model): 79%	Prediction of AKI in DCDLT patients
Hu et al. [131]	2022	XGBoost model RF model NB model Log. Reg. model SVM model kNN model DT model	22,360 ICU patients from MIMIC-IV database, released by BIDMC’s ICU, Boston, MA, USA	29 (demographic information, clinical data, laboratory data, and previous pathologies)	AUC-ROC (XGBoost model): 89% Accuracy (XGBoost model): 87.70%	Prediction of mortality in ICU patients with AKI
Inaguma et al. [124]	2020	Log. Reg. model RF model	9911 CKD patients from Fujita Health University Hospital, Toyonaka, Aichi, Osaka, Kansai, Japan	11 (clinical data, laboratory data, and previous pathologies)	AUC-ROC (RF model): 73%	Prediction of the rapid renal decline in CKD patients
Islam et al. [75]	2019	AdaBoost model + J48 model LogitBoost model + Ant-Miner	2800 CKD and non-CKD patients from unspecified hospitals	24 (demographic information, clinical variables, laboratory data, and previous pathologies)	F_1_-Score (LogitBoost + Ant-Miner models): 99.75%	Prediction of CKD patients and rule generation of the relationship between CKD attributes
Kalisnik et al. [111]	2022	Detect-A(K)I model Log. Reg. model RF model SVM model DNN model	7214 cardiac surgery patients from the Department of Cardiac Surgery at Klinikum Nurernberg-Paracelsus Medical University, Nurnberg, Germany	21 (demographic information, pre-operative clinical status, peri-operative, and post-operative variables)	Accuracy (Detect-A(K)I model): 82.10% AUC-ROC (Detect-A(K)I model): 88%	Early detection of CSA-AKI patients
Kanda et al. [93]	2019	BNN model + SVM model	7465 health patients from Yamagata, Tohoku, Japan	11 (demographic information, clinical variables, and laboratory data)	Generic test error (unspecified): 0.1186	Identification of patients at risk of CKD progression in a healthy population
Kanda et al. [118]	2022	XGBoost model Log. Reg. model DNN model	24,949 adult hyperkalemic patients the Japanese national database Medical Data Vision Company, Tokyo, Japan + 86,279 adult hyperkalemic patients from the Japanese national database Real World Data Vision Company, Osaka, Japan	64 (medications, medical history, and risk factors)	AUC-ROC (XGBoost model): 95.70%	Prediction of CKD development in hyperkalemic patients
Kandasamy et al. [127]	2023	GMM model + Hybrid RBFNN model	40,000 CKD patients from MIMIC-IV database, released by BIDMC’s ICU, Boston, MA, USA	Laboratory results, radiographies, clinical notes and observations, progress reports, historical medication records, and patients’ personal information	n.a. (framework designed to exceed the performance of existing models)	Prediction of disease progression in CKD patients
Kers et al. [141]	2022	CNN model	5844 digital WSIs of kidney allograft biopsies from 1948 patients hospitalized in Amsterdam University Medical Center, Amsterdam, Netherlands; from the University Medical Center of Utrecht, Utrecht, Netherlands; and from the Institute of Pathology, Rheinisch-Westfälische Technische Hochschule (RWTH) Aachen University, Aachen, Germany	Structural and morphological features extracted by the model	AUC-ROC: ≃78%	Classification of kidney allograft biopsies into normal and rejected to predict kidney rejection in transplant patients
Konieczny et al. [115]	2021	RF model MLP model DT model Gaussian NB model AdaBoost model SVM model kNN model	80 patients with biopsy-proven IgAN from the Department of Nephrology and Transplantation Medicine, Wroclaw Medical University, Wroclaw, Poland	35 (demographic information, clinical variables, and laboratory data)	Accuracy (RF model): 80.25%	Prediction of renal functions deterioration in patients with IgA nephropathy
Kordzadeh et al. [108]	2021	ANN model	241 post-EndoVascular Aneurysm Repair (post-EVAR) patients from Mid Essex National Health Service (NHS) Trust Foundation, Essex, UK	26 (demographic information, pre-operative variables, post-operative complications, aneurysm morphology, and hospitalization data)	Accuracy: >86%	Prediction of possible complications post-EVAR in operated patients
Kuo et al. [126]	2019	ResNet model XGBoost model	4505 kidney UltraSound (US) images of 1299 patients from China Medical University Hospital, Taiwan	Structural and morphological features extracted by the model	Accuracy (ResNet model): 85.60% AUC-ROC (ResNet model): 90.40%	Prediction of renal functions deterioration and its severity based on patients’ eGFR values
Lakshmanaprabu et al. [90]	2019	PSO algorithm + DNN model	400 records from the ML repository of the University of California, Irvine, CA, USA	24 (demographic information, clinical variables, and laboratory data)	AUC-ROC: 98.47% Accuracy: 99.25%	Prediction and severity assessment of CKD patients using IoT and cloud-based frameworks
Lee et al. [114]	2022	RF model Extra Trees model XGBoost model Landmark-Boosting model LGBM model Log. Reg. model	11,661 CKD patients from Taipei Veterans General Hospital (VGH) Big Data Center, Taipei, Taiwan	Demographic characteristics, comorbidities, laboratory data, and medication prescriptions	AUC-ROC (Landmark-Boosting model): 87.90% Accuracy (Landmark-Boosting model): 89.10%	Prediction of ESKD development in CKD patients surviving sepsis
Lee et al. [123]	2022	Transfer learning with pre-trained neural networks + BoW method + clustering algorithm + RF model	107,471 histopathology images obtained from 161 biopsy of 57 patients, released by C-PROBE cohort, University of Michigan, MI, USA	4 (demographic information, and clinical variables)	AUC-ROC: 93% (present), 80% (1 year apart) Accuracy: 90.17% (present), 78.27% (1 year apart)	Prediction of the kidney function and 1-year change based on patients’ eGFR values
Lei et al. [99]	2019	Log. Reg. model GBM model RF model	42,615 surgical patients from four academic hospitals, USA	Pre-hospitalization, pre-operative, and peri-operative variables	AUC-ROC (GBM model): 81.70%	Prediction of post-operative AKI in patients undergoing surgery
Liu et al. [132]	2022	DT model RF model SVM model kNN model Log. Reg. model	2678 HF patients from MIMIC-IV database, released by BIDMC, Boston, MA, USA	39 (demographic information, clinical variables, laboratory data, and medications)	AUC-ROC (RF model): 96%	Prediction of AKI occurrence in heart failure patients
Liu et al. [109]	2023	SVM model Log. Reg. model RF model kNN model LGBM model XGBoost model	88 post kidney transplantation patients with pneumocystis carinii pneumonia from Renmin Hospital of Wuhan University, Wuhan, China	5 (demographic data, clinical manifestations upon admission, laboratory results, and past medical history)	AUC-ROC (RF model): 92%	Prediction of severe pneumocystis carinii pneumonia in post kidney-transplant patients
Lokuarachchi et al. [117]	2020	ANN model (1) CNN model (2) RF model (3)	609 CKDu patients from Care Research Center of Panadura, Panadura, Sri Lanka	22 (1) (clinical variables, laboratory data, and risk factors) n.a. (2) n.a. (3)	R^2^ Score (ANN model): 0.143 Specificity (CNN model): 89.28% n.a. (RF model)	Prediction of CKDu patients considering creatinine level of blood (1), Kidney Disease Quality of Life (KDQOL) score (1), ankle swelling (2), and risk factors influencing creatinine level of blood (3)
Lu et al. [113]	2022	LSTM model + FNN model	4839 AKI and non-AKI hospitalized patients with COVID-19 from Montefiore Health System, New York City, NY, USA; and from Stony Brook University Hospital, New York City, NY, USA	19 (demographic information, clinical variables, laboratory data, and comorbidities)	AUC-ROC: 96.50% Accuracy: 89.57%	Prediction of AKI onset in hospitalized patients with COVID-19
Marechal et al. [94]	2022	Combination of two CNN models	241 samples from patients with healthy kidney tissue from University Hospital of Dijon, Dijon, France; and from the University Hospital of Besancon, Besancon, France	Glomerular density, glomerular volume, vascular luminal stenosis, severity of interstitial fibrosis or tubular atrophy	Accuracy: >90% AUC-ROC: 92%	Prediction of CKD in healthy patients analyzing histological prognostic factors
Mathis et al. [105]	2020	Combination of two multivariate logistic regression models	138,021 patients underwent non-cardiac interventions from eight academic and private centers, USA	Demographic information, clinical variables, pre-operative, and operative data	Odds ratio *intraoperative hypotension—AKI patients*: 2.62 C-index: 73%	Prediction of AKI risk linked to the intraoperative hypotension in patients underwent non-cardiac interventions
Moreno-Sànchez [84]	2023	RF model Extra Trees model XGBoost model AdaBoost model	400 records from Apollo Hospitals, Karaikudi, India, released by the ML repository of the University of California, Irvine, CA, USA	25 (demographic information, clinical variables, and laboratory data)	F_1_-Score (XGBoost model): 99.40% Accuracy (XGBoost model): 97.50%	Early diagnosis of CKD patients using an explainable model
Naser et al. [112]	2021	Multivariate logistic regression model	353 COVID-19 patients from Bahrain Defence Force (BDF) Royal Medical Services, Riffa, Bahrain	Demographic information, laboratory test data, and medical conditions	Odds ratio *AKI patients died—no AKI patients*: 48.6	Prognosis of AKI in patients affected by COVID-19
Nasir et al. [72]	2022	Transfer learning with pre-trained AlexNet model + SGDM algorithm + ADAM algorithm + RMSPROP algorithm	3300 data samples images of kidney cancer from the online source Kaggle database, released by Google Limited Liability Company (LLC), Mountain View, CA, USA	Structural and morphological features extracted by the model	Accuracy: 99.20% F_1_-Score: 99.70%	Early diagnosis of renal cancer patients using a combination of IoT and blockchain technologies for data security
Nunez et al. [85]	2022	SVM model kNN model NB model	400 records from the ML repository of the University of California, Irvine, CA, USA + kidney diseases data from the online source Kaggle database, released by Google LLC, Mountain View, CA, USA	25 (demographic information, clinical variables, and laboratory data)	Accuracy (NB model): 96%	Early diagnosis of CKD patients analyzing relevant risk factors
Ogunleye et al. [78]	2020	Log. Reg. model LDA model SVM model kNN model XGBoost model	400 records from the ML repository of the University of California, Irvine, CA, USA	25 (demographic information, clinical variables, and laboratory data)	Accuracy (XGBoost model): ≃100% AUC-ROC (XGBoost model): ≃100%	Early diagnosis of CKD patients based on their data
Ou et al. [142]	2023	Log. Reg. model Extra Trees model RF model GBDT model XGBoost model LGBM model	53,477 diabetic patients from Taipei Veterans General Hospital, Taipei, Taiwan	78 (demographic information, medications, previous pathologies, and laboratory data)	AUC-ROC (XGBoost model): 95.30%	Prediction of ESKD in newly diagnosed diabetic patients based on their routine data
Paquette et al. [106]	2022	DeepSurv model DeepHit model RF model RNN model	180,141 transplant patients from Scientific Registry of Transplant Recipients (SRTR), released by Hennepin Healthcare Research Institute, Minneapolis, MN, USA	170 (socio-demographic information, previous pathologies, laboratory data, and transplant details)	C-index (DeepHit): 66.10%	Prediction of graft survival probability after kidney transplantation from deceased donors
Pareek et al. [69]	2023	CNN model	n.a.	25 (demographic information, laboratory data, and clinical variables)	F_1_-Score: 96%	Prediction of early stages of CKD based on patients’ data
Patel et al. [87]	2022	XGBoost model DT model AdaBoost model SVM model kNN model Log. Reg. model RF model NB model	400 records from the ML repository of the University of California, Irvine, CA, USA	25 (demographic information, clinical variables, and laboratory data)	F_1_-Score (RF model, Log. Reg. model): 99% F_1_-Score (RF model, Log. Reg. Model): 99%	Prediction of CKD based on patients’ data
Pati et al. [89]	2023	ANN model + Voting technique ANN model + Bagging technique ANN model + Voting technique + Bagging technique	400 records from Apollo Hospitals, Tamil Nadu, India, released by the ML repository of the University of California, Irvine, CA, USA	25 (demographic information, clinical variables, and laboratory data)	F_1_-Score (ANN model + Voting Classifier + Bagging Classifier): 98.80% Accuracy (ANN model + Voting Classifier + Bagging Classifier): 96.67%	Prediction of CKD based on patients’ data
Poonia et al. [80]	2022	Log. Reg. model + Chi-square algorithm kNN model ANN model SVM model NB model	400 records from the ML repository of the University of California, Irvine, CA, USA	24 (demographic information, clinical variables, and laboratory data)	Accuracy (Log. Reg. model): ≃98% F_1_-Score (Log. Reg. model): ≃98%	Prediction of CKD considering the most relevant patients’ data
Priya et al. [83]	2023	ANN model SVM model kNN model DT model (all post-processed with the HGSAPSO algorithm)	400 records from the ML repository of the University of California, Irvine, CA, USA	25 (demographic information, clinical variables, and laboratory data)	Accuracy (ANN model + HGSAPSO algorithm): 93%	Prediction of CKD based on patients’ data
Rabby et al. [74]	2019	kNN model SVM model RF model Gaussian NB model AdaBoost model LDA model Log. Reg model DT model GBM model ANN model	400 records of South Indian patients from the ML repository of the University of California, Irvine, CA, USA	25 (demographic information, clinical variables, and laboratory data)	Accuracy (DT model, Gaussian NB model): ≃100% F_1_-Score (DT model, Gaussian NB model): ≃100%	Prediction of CKD based on patients’ data
Rady et al. [135]	2019	PNN model MLP model SVM model RBFNN model	361 CKD Indian patients from the ML repository of the University of California, Irvine, CA, USA	25 (demographic information, clinical variables, and laboratory data)	Accuracy (PNN model): ≃99% F_1_-Score (PNN model): ≃97%	Prediction of CKD patients and classification in 5 stages based on eGFR values
Rajeshwari et al. [81]	2022	NB model RF model DT model SVM model	400 records of 80 CKD and non-CKD Indian patients from unspecified hospital structures in India	14 (clinical variables, and laboratory data)	F_1_-Score (RF model): 99% Accuracy (RF model): 98.75%	Prediction of CKD based on patients’ data
Rashid et al. [82]	2022	Log. Reg. model NB model kNN model SVM model RF model DT model PSO algorithm + ANN model	189 CKD patients from the ML repository of the University of California, Irvine, CA, USA + online source Kaggle database, released by Google LLC, Mountain View, CA, USA + online source Dataworld database, Austin, TX, USA	26 (demographic information, laboratory data, and previous pathologies)	Accuracy (PSO algorithm + ANN model): 99.76%	Prediction of CKD considering the most relevant patients’ features
Rashidi et al. [97]	2020	Log. Reg. model kNN model RF model SVM model DNN model	50 patients with burns major than 20% of the total body + 51 patients with injuries unrelated to burn trauma, both from the University of California Davis Health Clinic Hospital, Sacramento, CA, USA	4 (laboratory data)	AUC-ROC (DNN model): 92% Accuracy (DNN model): 92%	Early identification of AKI in burned and non-burned trauma patients
Raynaud et al. [107]	2021	DISPO model	13,608 transplant patients from eighteen academic transplant centers in Europe, the United States, and South America	eGFR and proteinuria measurements + clinical, histological, and immunological variables	AUC-ROC: 85.70%	Prediction of renal survival among kidney transplant recipients
Revathi et al. [86]	2023	ANN model NB model kNN model SVM model DT model Log. Reg. model	400 records of South Indian patients from the ML repository of the University of California, Irvine, CA, USA	25 (demographic information, clinical variables, and laboratory data)	F_1_-Score (ANN model): 96% Accuracy (ANN model): 96%	Prediction of CKD based on patients’ data
Schena et al. [116]	2021	ANN model (classification model + regressor model)	1115 IgAN patients from the European Validation Study of the Oxford Classification of IgAN (called “VALIGA”) cohort; from Thessaloniki Renal Unit, Greece; and from six undefined renal units in Europe	7 (demographic information, clinical variables, laboratory data, disease status, and therapy)	AUC-ROC: 82% (5 years apart), 89% (10 years apart)	Prediction of ESKD and time remaining to its onset in patients with primary IgAN
Schmid et al. [101]	2023	Undefined ML model	21,045 ICU patients from Robert Bosch Hospital, Stuttgart, Germany	Demographic information, clinical variables, and laboratory data	1.80% of AKI cases documented vs. 65.40% automatically detected	Prediction of AKI in post-operative patients
Schulz et al. [70]	2021	MMDLM model	230 ccRCC patients from the TCGA database, Germany + 18 patients from the University Medical Center of Mainz, Mainz, Germany	Structural and morphological features extracted by the model	Accuracy: 83.43% AUC-ROC: 91.60% AUC-PR: 94.40% C-index: 81.23%	Prediction of prognosis in ccRCC patients
Shabaniyan et al. [139]	2019	QDA model kNN model MLP model SVM model (all preceded by SFS + FDA algorithms)	254 PCNL and CKD patients from Faqihi Hospital, Shiraz, Iran	26 (demographic information, renal stones characteristics, previous pathologies, and laboratory data)	Accuracy (SFS + FDA algorithms + SVM model): 94.80%	Prediction of post-PCNL treatment outcomes in patients with large kidney stones
Song et al. [119]	2020	Landmark-Boosting model	14,039 adult patients with type 2 diabetes from the Healthcare Enterprise Repository for Ontological Narration (HERON) clinical data repository of the University of Kansas, KS, USA	6624 (visit details, procedures, laboratory test, medications, allergies, diagnoses, alerts, and demographic data)	AUC-ROC: 83% (years 2 since diabetes mellitus onset) AUC-PR: 75% (years 4 since diabetes mellitus onset)	Prediction of CKD among patients with type 2 diabetes
Song et al. [125]	2020	DS-GBT model	153,821 hospital admissions from twelve independent health systems of the Greater Plains Collaborative network, USA	1933 (demographic, clinical, hospital encounter, and outcome variables)	AUC-ROC: ≃81%	Prediction of patients at risk of developing AKI among three possible stages within 48 h of hospital admission
Tomašev et al. [133]	2019	SRU model	703,782 hospitalized adult patients from more than 1200 sites at Department of Veterans Affairs (VA), USA	315 (demographic and admission information, clinical variables, laboratory tests, and diagnoses)	AUC-ROC: 92.10%	Risk prediction of future deterioration in hospitalized adult patients up to 48 h in advance
Tran et al. [138]	2023	BNN model DL model Log. Reg. model RF model	534 CKD patients from Photo-Graphe 3 Study, France	7 (demographic information, previous pathologies, laboratory data, and nutritional status)	AUC-ROC (Log. Reg. model): 76% Accuracy (Log. Reg. model): 81.80%	Prediction of 2-year mortality in end-stages CKD patients
Tseng et al. [110]	2020	Log. Reg. model SVM model RF model XGBoost model RF model + XGBoost model	671 patients undergoing cardiac surgery from Far Eastern Memorial Hospital (FEMH), New Taipei City, Taiwan	94 (demographic information, clinical variables, pre-operative, and intraoperative variables)	AUC-ROC (RF model + XGBoost model): 84.30%	Prediction of mortality in CSA-AKI patients
Wang [76]	2020	Log. Reg. model DNN model	400 records of kidney disease patients from undefined hospitals in India	25 (demographic information, clinical variables, and laboratory data)	F_1_-Score (DNN model): 95% AUC-ROC (DNN model): 96%	Prediction of CKD based on patients’ data
Wessels et al. [120]	2023	DINO-ViT model	709 renal WSIs from the TCGA, USA + University Medical Centre of Mannheim, Mannheim, Germany	Structural and morphological features extracted by the model	Hazard ratio *disease survival*: 2.31	Prediction of disease-specific survival in ccRCC patients using histopathological images
Xiao et al. [134]	2019	ElasticNet model LASSO model Ridge model Log. Reg. model SVM model RF model XGBoost model kNN model NN model	551 patients with proteinuria from the Department of Nephrology at Huadong Hospital, Shanghai, China, affiliated with Fudan University, Shanghai, China	18 (demographic information, and laboratory data)	AUC-ROC (Log. Reg. model): 87.30%	Prediction of proteinuria severity and progress in CKD patients
Xu et al. [122]	2020	kNN model Log. Reg. model RF classifiers DT model	1117 EHRs of patients with type 2 diabetes from Beijing Pinggu Hospital, Beijing, China	29 (demographic information, and laboratory data)	Accuracy (RF classifiers): 89.15% F_1_-Score (RF classifiers): 94%	Early diagnosis of diabetic kidney disease patients and identification of risk groups
Yue et al. [98]	2022	Log. Reg. model kNN model SVM model DT model RF model XGBoost model ANN model	3176 patients affected by sepsis from MIMIC-III database, released by BIDMC, Boston, MA, USA	36 (demographic information, previous pathologies, laboratory data, therapy, and vital signs)	AUC-ROC (XGBoost model): 81.70% Accuracy (XGBoost model): 83.20% F_1_-Score (XGBoost model): 89.50%	Prediction of AKI development in patients with sepsis
Zhang et al. [103]	2021	Log. Reg. model SVM model RF model GBM model AdaBoost model	975 patients underwent liver transplantation from the Third Affiliated Hospital of Sun Yat-sen University-Lingnan Hospital, Guangdong, China	14 (demographic information, peri-operative variables, donor characteristics, etiology, previous pathologies, and medications)	AUC-ROC (GBM model): 76% F_1_-Score (GBM model): 73%	Prediction of AKI patients after liver transplantation
Zimmerman et al. [128]	2019	Multivariate logistic regression model RF model ANN model	23,950 ICU patients from MIMIC-III database, released by BIDMC, Boston, MA, USA	22 (demographic information, laboratory data, and vital signs)	AUC-ROC (Multivariate logistic regression model): ≃78%	Prediction of AKI onset in ICU patients within 72 h of hospital admission

### 3.2. Machine Learning as a Tool to Detect Kidney Diseases

People with KDs should have regular checks of their kidney function and blood pressure. Urine tests for blood or protein losses, blood tests, or kidney scans are just some of the tests useful for their detection [143]. The identification of kidney diseases at early stages can calm the pathology for further growth and restrain the difficult situations or complications on the patient’s health condition [144].

As in the case of prediction, and also in the detection case, the use of intelligent systems, such as AI tools, can save many lives.

Initial detection and therapy are indeed the best tools for combating chronic kidney disease, and several machine learning models proposed in the literature, such as support vector machine, k-nearest neighbors, decision tree, gradient boosting machine, and random forest, could be an important aid for clinicians’ assessment of the disease.

These tools can act on numerical data or images, for which nephron-pathologists and computer scientists have made considerable progress in developing models able to identify histological structures within WSIs, quantify histological structures, and classify diseases.

In this section, the most recent works dealing with KDs detection with artificial intelligence tools are reviewed.

Interesting applications of smart machine learning models in the renal field concerned the diagnosis of the presence of kidney diseases in pathological patients [145,146,147,148,149,150,151]. Accuracy values up to approximately 100% were obtained and models, such as artificial neural network [148], random forest [145], multilayer perceptron [150], and J48 decision tree [146], that were able to handle unbalanced and missing data better than a classic DT, and a Self-Attention Convolutional Neural Network (SACNN) [151], were implemented. Specifically, “self-attention” is a mechanism that allows the models to assign different importance to each element of an input than to all other elements in the set. Combined with the Season Optimization Algorithm (SOA), which enhances the accuracy and reduces the computation time of AI models, SACNN allowed the authors to achieve the high result mentioned above.

Among the models mentioned, the best result was achieved by Khamparia et al. [147] with the development of an artificial intelligence model capable of identifying healthy and unhealthy patients among 400 individuals. The model applied was a neural network given by the combination of a multilayer autoencoder and the SoftMax probabilistic classifier, used, respectively, for extracting useful features from the dataset and for the final classification, carried out by transforming the raw values into probabilities. The lowest accuracy values were instead obtained by Alsadi et al. [149], who achieved a kidney disease detection accuracy of 78% by analyzing immunobiological materials in kidney biopsies with CNN models. Specifically, they used several versions of pre-trained CNN models on a big dataset of natural images called “ImageNet”, such as ResNet, Inception-v3, Inception-Residual Network version 2 (InceptionResNet-v2), Neural Architecture Search Network (NASNet), and Visual Geometry Group with 16 and 19 convolutional layers (VGG-16/19), and identified the best model. VGG-19 is a model with a simple architecture consisting of many convolutional layers followed by fully connected layers, capable of accurately recognizing specific portions of the images, but taking a lot of time and computing power.

Despite the complexity of this model, its low accuracy could be attributed to the use of Electron Microscopy (EM) images, which, compared to CT or US images, contain intricate ultrastructural details that can closely resemble artifacts or non-pathological structures, potentially confounding the model’s ability to make accurate distinctions.

Beyond these challenges, some authors have implemented models able to detect not only renal diseases, but also the correct intake of potassium, as performed by Granal et al. [152] with the development of a Bayesian-based neural network applied to a dataset of 375 patients with CKD.

As in the predictive case, ML tools have focused on the detection of chronic kidney disease through image analysis or segmentation. Image classifiers or segmentation models were developed by different researchers [153,154,155,156,157,158,159,160], obtaining rapid and precise CKD detection.

The increasingly abundant availability of images prompted the researchers to use CNNs for almost all of these studies, such as ResNets [156,157], or the innovative Single Shot Detector (SSD) Inception-v2, a model implemented by Onthoni et al. [154] to locate and classify multiple objects in an image with a single pass through the network. U-Nets have also been used, either alone [158] or also supported by algorithms as the Extended Maxima Transform (EMT) and Slice Scanning (SSA), able to refine and optimize the detection of kidney boundaries, as in the case of Les et al. [155]. A non-convolutional exception was the hybrid fuzzy-deep neural network implemented by Kumar et al. [160] to recognize kidney disease through image processing. Regardless of the algorithm used, the choice of all these models proved to be optimal, as high values of F_1_-Score (89.30% [155], 94% [158], 98.70% [159]), accuracy (>90% [153], 92.90% [156], 99.23% [160]), mAP (94% [154]), and AUC-ROC (98% [157]) were obtained.

Other models were implemented to detect specific pathologies, such as Membranous Nephropathy (MN) [161], kidney stones [162,163,164,165,166], kidney cancer [167], or both [168], and Interstitial Fibrosis and Tubular Atrophy (IFTA) [169,170,171].

Computational platforms available online [167], CNNs optimized with artificial data augmentation and dropout [166], an algorithm that “turns off” some neurons to avoid the overfitting during the training phase, and CNN subcategories [171] such as U-Net [170], ResNet [163,168], or their combination [164] were developed to analyze CTs, US images, or WSIs. Specifically, Li et al. [164] identified ResU-Net as the best model following a comparison between specific transformers designed for semantic segmentation of high-resolution images, such as U-NEt TRansformer (UNETR), or multiple convolutional neural networks designed for this task, such as SegNet or DeepLab-v3, identified instead by Ginley et al. [169] as a good segmentation model if combined with a ResNet model, achieving up to 89% accuracy.

**Figure 13:**
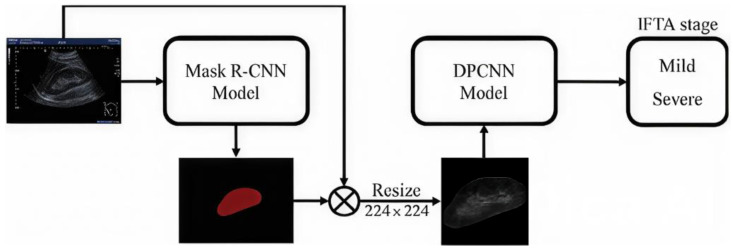
Flowchart of Mask Region-based Convolutional Neural Network (Mask R-CNN) and Dual-Path Convolutional Neural Network (DPCNN) proposed for Interstitial Fibrosis and Tubular Atrophy (IFTA) stage by Ginley et al. [169]. The arrow starting from the input image without going through the Mask-CNN indicates that the image can be analyzed directly by the DPCNN. This approach can simplify and speed up the process, but it isolates the kidney with less precision. Reproduced from Tang et al. [171].

On the other hand, Tang et al. [171] used a combination of Mask Region-based Convolutional Neural Network (Mask R-CNN) and Dual-Path Convolutional Neural Network (DPCNN), developed respectively for segmentation of the kidney region under investigation and extraction of features of interest from segmented and resized US images. Figure 13 shows the proposed AI prediction model for IFTA detection.

Due to its “duality”, the DPCNN was able to identify low-level and high-level features on detailed masks provided by Mask R-CNN, an algorithm able to segment, unlike classical CNNs, at the pixel level. Given its high performance, Mask R-CNN was also used by Yi et al. [170] on images segmented by U-Net to improve the localization and classification of abnormal renal structures.

Studies cited also include GrayNet-SB, a Surface-Based (SB) model implemented by Parakh et al. [162] by pre-training a CNN on the GrayNet online medical database, which performed better than ImageNet-SB, a CNN pre-trained on the ImageNet database, and RandomNet-SB, a CNN trained only on the 535 abdomino-pelvic CT scans selected by the authors.

Innovative models called “StoneNet” and “MN-Net” were proposed by Asif et al. [165] and Hao et al. [161], respectively. Developed for the detection of kidney stones, StoneNet was presented as a novel model based on MobileNet, a CNN architecture designed to be lightweight and suitable for devices with limited computational resources, such as mobile phones. On the other hand, as the name suggests, MN-Net was proposed to detect membranous nephropathy in renal pathological images, a kidney disease that affects the glomeruli. Specifically, Membranous Nephropathy Network (MN-Net) was implemented by combining YOLO-v3, a CNN model useful for detecting glomeruli within the images, and a glomeruli classification network based on Multiple Instance Learning (MIL), a machine learning paradigm applied in contexts where precise data labeling is not available at the individual instance level but only at the group (or bag) level, as in this case.

All these studies demonstrate the great interest in the search for an accurate diagnostic method of kidney diseases, which may lead to a serious deterioration in the renal activity of patients, up to transplantation in the worst cases.

High performances were indeed obtained in many of the models mentioned, such as accuracy values close to or higher than 97% [163,164,165,166], or F_1_-Score equal to or higher than 95% [161,162,168].

Particular interest has also been directed towards diseases involving glomeruli [172,173] to identify sometimes specific pathologies such as glomerulosclerosis [169,174,175] or glomerular lesions [176,177]. Specifically, glomerulosclerosis is a kidney disease that involves scarring of the glomeruli, the microscopic kidney units responsible for filtering blood to produce urine. In severe cases, this disease may lead to chronic renal failure. Prominent among these models is the U-Net used by Zhang et al. [173] in combination with a convolutional Multiple-Attention Network (MANet). The concept of multiple attention in deep learning refers to a mechanism that allows the model to focus on specific parts of the input during processing. Such a sophisticated approach allowed the authors to achieve 98% accuracy in classifying glomerular pathologies from immunofluorescence images. Another interesting approach was adopted by Lu et al. [174], who implemented Big Transfer-Small (BiT-S) and Big Transfer-Medium (BiT-M) pre-training, respectively, a ResNet model on ImageNet and ImageNet-21k, another large dataset of natural images similar to ImageNet. Specifically, the use of BiT-M proved to be particularly effective in the detection and classification of glomerulosclerosis, achieving an AUC-ROC of 99.40% and demonstrating how transfer learning is highly effective in improving the performance of deep learning models on complex and specific classification tasks, especially when only a limited amount of data is available in the target domain.

Among the cited works, an unpublished model was proposed by Nan et al. [177], i.e., the Uncertainty-Aided Apportionment Network (UAAN) associated with a SegNet model to segment, recognize, and classify glomerular lesions from WSIs. UAAN is a particular classification neural network designed to address problems in which uncertainty in data or predictions can prevent effective prediction of conventional models. This type of network incorporates mechanisms to quantify and utilize uncertainty, thereby improving model decisions and predictions. With the implementation of this model, anticipated by a segmentation optimization algorithm called “Focal Instance Structural Similarity” (FISS), the authors achieved an accuracy of 95.17%, outperforming traditional models such as ResNet, DenseNet, and InceptionNet models, which achieved accuracy values of no more than 86.35%.

Good results were also obtained by other authors, such as an accuracy of 90% reached by Akatsuka et al. [176] using a DenseNet model or an accuracy of 99% obtained by Pesce et al. [175] using a feature-based ANN, a slower but slightly better performing model of Watson Visual Recognition (WVR), which, unlike the former, analyzes and classifies images directly without requiring manual feature extraction. An accuracy of 98.62% was obtained with the WVR model.

Identifying the presence of chronic kidney disease can also be achieved through precise analysis of relevant substances in the renal field. In this regard, some important factors are extracellular calcium and already mentioned potassium ionic concentrations [178], albuminuria levels [179], or urea levels in the saliva sample [180]. Artificial neural networks [178], the combination of deep and not deep algorithms [180], or the use of rule extraction algorithms DT-based as continuous Recursive Rule eXtraction (Re-RX) to make deep models interpretable in presence of continuous variables [179], performed well in terms of accuracy (77.56% [179], 98.04% [180]) or test error (0.01, [178]).

A novel approach using images was finally presented by Sabanayagam et al. [181] and Zhang et al. [182], who developed deep learning algorithms alone [182] or supported by clinical risk factor analysis models [181] to detect chronic kidney disease from retinal fundus photographs, obtaining AUC-ROC values exceeding 81%. Detection of CKD is possible because deteriorating kidney function causes retinal problems such as vascular endothelial dysfunction, chronic inflammation, and thrombosis status, which are easily detected with artificial intelligence tools for image analysis. By developing this type of AI model, the authors in ref. [181,182] have thus contributed to a potential improvement in the complex process of diagnosing kidney disease.

Table 5 reports a list of all quoted literature works, providing key details on adopted models, a brief description of the dataset employed and their origin, the number and type of features used to train the model, the results, in terms of performance parameters, obtained by the best performing model among those tested, and in the last column, the purpose of the work.

### 3.3. Machine Learning as an Assistant Tool for Nephrologists

The number of patients with end-stage kidney disease needing renal replacement therapy is estimated to be between ~4.9 and ~7.1 million [64] globally.

Dialysis is certainly a life-saving therapy for patients with end-stage kidney disease, but there are many problems that can affect them, such as maintaining a stable volume or pressure during dialysis sessions or the onset of dialysis disorders, such as cramps.

New approaches based on artificial intelligence techniques can identify volume-related adverse events, hypotension, or general discomfort during the treatment.

These are just some of the many applications of artificial intelligence as a tool to assist nephrologists in the complex management of hemodialysis therapies.

IntraDialytic Hypotension (IDH) is the main complication of hemodialysis treatment, since it affects more than 10% of patients undergoing treatment [183]. Many authors have addressed this problem using artificial intelligence models, attempting to predict IDH [184,185,186,187] and other minor dialysis discomfort [188,189] at the start of therapy on the basis of clinical and analytical data from patients. These findings collectively highlighted that blood pressure trends, ultrafiltration rate, and patient comorbidities are the strongest predictors of IDH, indicating that future AI-based tools should integrate both static patient characteristics and dynamic intradialytic signals to optimize therapy. Using different approaches, from the simplest machine learning models [185,189] to the most complex neural networks [186,188], part of these studies resulted in accuracy [186,188,189] or AUC-ROC [185] values of over 96%. Particularly interesting is the model bCOWOA-KELM [184], where the “binary COvariance” (bCO) matrix-driven version of the Whale Optimization Algorithm (WOA) was used to select better than other optimization algorithms the most relevant features for the Kernel Extreme Learning Machine (KELM), a type of feedforward neural network with a single hidden layer capable of handling non-linear data. Although it achieved a slightly lower accuracy, equal to 92.41%, this model turns out to be the most structurally complex among those proposed. Relevant is also the “Robust Deep Learning-based Clinical Data Classification” (RDLCDC), developed by Mohammed et al. [186], optimizing a CNN with the Sparrow Search Optimization (SSO), an algorithm able to adjust critical parameters related to the structure of AI models. Given its specific application, the model was more precisely named RDLCDC-IDH, with which the authors obtained an IDH diagnostic accuracy of 97.70%.

The high performance obtained by these models demonstrates their potential to serve as a valuable aid in the management of intradialytic hypotension, provided that future studies focus on improving their generalizability and transparency, thereby facilitating safe and trustworthy clinical deployment.

A second major problem in hemodialysis therapies concerns the atherosclerotic fistula, which is often subject to aneurysms, systoles, or infections due to the continuous stress it undergoes during treatment. Some authors have, therefore, developed models capable of diagnosing such problems by analyzing audio recordings [190,191], patients’ data [192], or images [193,194]. Specifically, using audio recordings, Song et al. [190] developed a ResNet combined with an ANN to non-invasively diagnose stenosis in arteriovenous fistulas, while Ota et al. [191] used a CNN in combination with LSTM and GRU to evaluate and classify arteriovenous bruits. Both achieved good results, more than 90% in F_1_-Score for the first case, and up to 92% in AUC-ROC value for the second one. The use of clinical and demographic data combined with models such as XGBoost, RF, and multiple regression then allowed Balamuthusamy et al. [192] to predict with an accuracy of 86% the risk of stratification and re-interventions on arteriovenous access, which passed from 1.8 to 1.1 per patient for one year. The problem of arteriovenous aneurysms or other complications, such as nerve injury, hematoma, or emphysema, was, respectively, addressed by Zhang et al. [193] and Hong [194], who used CNNs for their optimal classification and care. These studies demonstrated how early and accurate detection of access dysfunction could allow nephrologists to intervene before thrombosis or infection occurs, potentially extending fistula lifespan and reducing the need for re-interventions, an important component of optimizing long-term dialysis therapy.

To predict these potential complications, many researchers have also developed models capable of well predicting the values of important parameters in therapy, such as Kt/V, heart rate, and blood pressure [195] with ANNs, or classifying the values of serum creatinine [196] with simpler ML models. Specifically, the values of serum creatinine were classified by Brito et al. [196] with an accuracy over 95% on an unusual dataset, i.e., patients undergoing peritoneal dialysis treatment, using kNN-based algorithms as IBk and kStar, or tree-based models as random forest, Reduced Error Pruning Tree (REPTree), and RandomTree. IBk and kStar are algorithms where the distance between values is calculated with the Euclidean distance and the entropy, respectively, while REPTree and RandomTree are single-tree algorithms optimized to improve generalization and reduce overfitting compared with standard decision trees.

Many authors have also developed AI models aimed at studying fluid overload [197] and atherosclerotic plaque formation [198] in hemodialyzed patients or the most significant parameters for screening the patients at high risk [199]. Among them, a particularly interesting study was presented by Tan et al. [197], who obtained comparable results using both YOLACT and Mask R-CNN on Lung UltraSound (LUS) images. Despite the simplicity of YOLACT, a model that detects and segments objects of interest in a single pass for the identification of the fluid overload, the authors achieved the same recall value of 83.30% obtained with the more complex Mask R-CNN model, which works in multiple passes to achieve the same goal. However, regardless of model complexity, both models clearly demonstrate that real-time fluid status assessment could support the implementation of adaptive ultrafiltration profiles, allowing fluid removal to be tailored to each patient’s hemodynamic tolerance and potentially minimizing cardiovascular stress.

**Figure 14:**
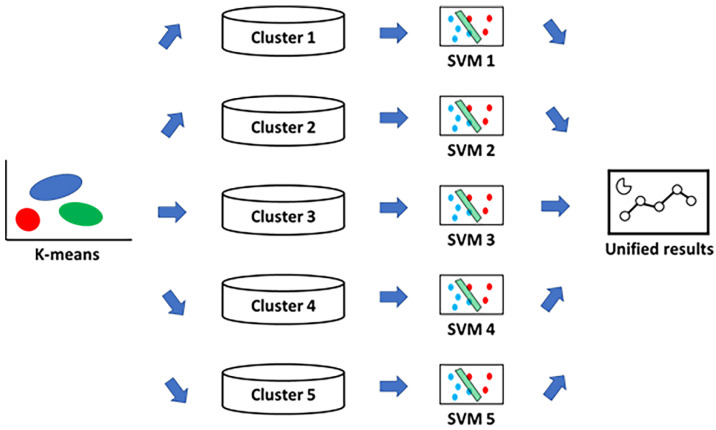
Structure of the ensemble model used to classify dialysis patients according to their risk level. Reproduced from Kanda et al. [199].

Equally innovative, Kanda et al. [199] analyzed hemodialysis patients in Japan by dividing them into clusters using the unsupervised k-means algorithm, where k represents the number of clusters. In their model, each cluster represented a distinct patient group with shared health profiles and potential risk factors, ensuring that patients within a cluster had more homogeneous health profiles. Each cluster was then analyzed with an SVM model for capturing and predicting the unique outcome patterns associated with each cluster’s characteristics more accurately than a single, unified model. The results of the individual SVM models were finally merged to generate an overall risk prediction for the entire patient population, taking advantage of the detailed analysis of each cluster. Figure 14 shows a graphical representation of this innovative ensemble model used by the authors.

Compared to simple machine learning models applied to a single dataset of mixed patients, this approach allowed the authors to obtain better results in classifying patients according to risk, achieving an accuracy of 94.80% and moving dialysis care closer to a precision-medicine paradigm.

Taken together, all the studies presented in this section therefore demonstrate that AI can not only predict adverse events but also serve as a decision-support tool to dynamically adjust treatment parameters, thereby optimizing both safety and efficacy of hemodialysis. Finally, one of the most popular topics in recent years concerns the use of chatbots for nursing [200] or patient care [201]. Specifically, Cheng et al. [201] implemented an AI ChatBot to be tested on patients at the PD center of the National Taiwan University Hospital in Taiwan. Providing valuable advice to dialysis patients at home, this ChatBot was a great success with the 440 participants, who were 90% satisfied at the end of the test. Qarajeh et al. [200] instead compared different ChatBots, such as ChatGPT, Bard AI, and Bing Chat, to classify foods according to their potassium and phosphorus content. Providing an accuracy of around 80%, this study demonstrated how these modern communication tools, if used correctly, can be really helpful to medical staff and patients themselves in the daily management of the disease, improving their quality of life.

Table 6 reports a list of all quoted literature works, providing key details on adopted models, a brief description of the dataset employed and their origin, the number and type of features used to train the model, the results, in terms of performance parameters, obtained by the best performing model among those tested, and in the last column, the purpose of the work.

### 3.4. Machine Learning as a Tool to Predict Long-Term Complications in Dialysis Patients

Another use of artificial intelligence algorithms in the dialysis field regards the prediction of the outcome of the therapy, days, months, or years later. Patients may experience many problems after multiple years of therapy, and careful monitoring of their condition can help to improve their quality of life. The onset of diseases such as anemia and sarcopenia, the prolonged hospital stays of dialysis patients, or their low probability of long-term survival are just some of these possible problems [202,203,204]. Specifically, sarcopenia is the progressive and generalized loss of muscle mass and strength, common in patients with chronic diseases. Anemia, on the other hand, is a condition in which the kidneys do not produce enough erythropoietin, the hormone that stimulates the production of red blood cells. Predicting these diseases can be important to avoid further complications in already compromised patients and permit preventive interventions such as nutritional support, exercise programs, and timely initiation of erythropoiesis-stimulating agents, ultimately helping to preserve functional status and improve quality of life. In this regard, there are numerous applications of AI models in the predictive-therapeutic field, which aim to analyze the clinical conditions of patients and anticipate possible future complications, including the most severe outcomes such as hospitalization or mortality.

Mortality in dialysis patients is a complex problem that can be influenced by several factors. Predicting it [205,206,207] or predicting the key factors responsible for it [208] has attracted the interest of several authors, who have studied the problem using different approaches. These studies not only contribute to understanding the problem but also offer potential solutions to improve patients’ quality of life and reduce mortality rates. Specifically, good and comparable AUC-ROC values were obtained, and several machine learning models were tested, such as the RF model [208] or the more sophisticated ANNs and DNNs [206,207]. Among these, the best predictive model was developed by Díez-Sanmartín et al. [205] using an XGBoost classifier optimized by clustering models and the so-called “False Clustering Discovery Reduction” (FCDR) algorithm, which refined the initial clustering models to reduce the total clusters distinguishing groups with statistically significant differences in risk profiles. By identifying high-risk subgroups, this type of modeling could enable clinicians to adjust dialysis prescriptions, manage comorbidities more aggressively, and potentially schedule closer follow-up for patients most likely to experience adverse outcomes. Implementing this model, an AUC-ROC of 99.09% was achieved.

Despite the slightly lower performance, the work of Noh et al. [206] is noteworthy, as they addressed the same task in terms of the C-index, a more reliable parameter for survival models, as it also considers the time variable, which is highly relevant for optimizing long-term treatment strategies. Comparing models as logistic regression, decision tree, random forest, Ridge, and LASSO, they found that the combination of LSTM and autoencoder exhibited the best performance in the 5-year prediction in terms of AUC-ROC, equal to 85.80%, but not in terms of C-index. The highest C-index, equal to 76.90%, was obtained using a Survival Tree, a DT model capable of predicting the time until an event, such as death. Similarly, Siga et al. [207] implemented an optimized Bayesian neural network capable of predicting patients’ conditions 2 years in advance with an AUC-ROC value of 78%, but without providing information on the C-index value. In its model, the optimization was achieved by building the Bayesian network with an algorithm called “Tree Augmented Naive” (TAN), which relaxed the BNN’s condition according to all variables must be conditionally independent given the output. This allowed the final model to capture more complex relationships between variables and improve its performance.

**Figure 15:**
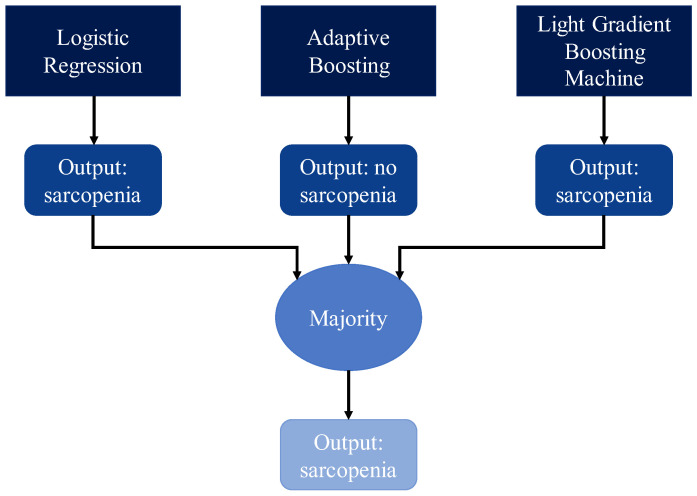
Representation of the voting classifier used by Liao et al. [210]. It combines the results of Logistic Regression (Log. Reg.), Adaptive Boosting (AdaBoost), and Light Gradient Boosting Machine (LGBM) models to improve the accuracy of the prediction, considering the “vote” cast by each model and the majority of votes obtained. In the figure, “presence of sarcopenia” is the majority output found by the models involved.

Other authors have developed ML models capable of predicting more specific problems, such as anemia [209], sarcopenia [210], or loss of arteriovenous fistula patency [211], very common in patients undergoing continuous dialysis treatment. All these studies provided good performance, up to an accuracy equal to 87% obtained by Ohara et al. [209] with their innovative model “Artificial Intelligence Supported Anemia Control System” (AISACS), given by the combination of a deep neural network and a recurrent neural network. Notably, comparing five binary machine learning methods and five tree-based models, Liao et al. [210] found that the voting classifier composed of Log. Reg., AdaBoost, and LGBM models guarantees the best performance in predicting sarcopenia at 3 days in men after the last hemodialysis treatment. Figure 15 presents the models selected to implement the voting classifier.

Using this approach for predicting sarcopenia in men, the authors obtained an AUC-ROC value of over 85%, but, for women, the best performing model was SVM, with an AUC-ROC of 77.69%. The features associated with sarcopenia in the female population are subtler and less distinct than in men, an aspect that made the SVM model the best among the classifiers tested, given its good ability to handle complex and nuanced relationships in the data [211].

Evaluating model performance using MAE, Garbelli et al. [211] reported promising predictive results with several classification methods. These included the BINary Classifier Model (BINCM), which uses XGBoost to simplify the problem into binary tasks, the Consistent Ordinal Regression Network (CORN), a neural network that works keeping proper class order, and a classical regression model called “REGM” by the authors, which employs XGBoost for analysis of continuous variables to be mapped into classes at the output. The high MAE values, up to 0.27 applying BINCM, demonstrated the potential of these approaches to improve AVF monitoring in clinical practice and how the integration of such models into routine surveillance protocols could allow earlier interventions, preserve vascular access longevity, and reduce costly surgical procedures.

Several authors have also analyzed the occurrence of COVID-19 in dialysis patients or the effects of vaccination cycles. In 2021, Monaghan et al. [212] developed a machine learning model capable of predicting the risk of contracting COVID-19 in HD patients at least 3 days before clinical suspicion of the disease. Using the XGBoost model and analyzing 40,490 patients, the model achieved an AUC-ROC value of 68%, which proved effective given the predictive complexity of the case. For predicting the survival of hemodialysis patients following COVID-19 vaccination, Tang et al. [213] compared several machine learning models, including LGBM, random forest, XGBoost, and CatBoost. They identified the best predictive model in the LGBM model, with an F_1_-Score of 95% achieved on a cohort of 433 HD patients in Taiwan, highlighting the potential of AI to inform infection control strategies and vaccination policies, which is particularly relevant for the highly vulnerable dialysis population.

Several authors have also developed ML models able to predict the prolonged hospital Length Of Stay (p-LOS) of dialysis patients caused by the onset of complications resulting from home dialysis treatments. As a matter of fact, the hospitalization rate is relatively high in patients undergoing peritoneal dialysis, and the management of those at high risk of p-LOS could improve the management of healthcare resources. In this regard, using simple machine learning models such as the RF model [214] or a stacking model given by the combination of multiple ML models [215], Wu et al. [214] and Kong et al. [215] both achieved AUC-ROC values of approximately 75%.

Altogether, all the studies of this section therefore demonstrate that predictive models can go beyond risk estimation to actively support therapeutic decision-making, allowing nephrologists to tailor interventions, allocate resources more efficiently, and improve long-term survival and quality of life.

Table 7 reports a list of all quoted literature works, providing key details on adopted models, a brief description of the dataset employed and their origin, the number and type of features used to train the model, the results, in terms of performance parameters, obtained by the best performing model among those tested, and in the last column, the purpose of the work.

### 3.5. Recent Developments and Emerging Trends in Artificial Intelligence for Nephrology

In recent years, research on artificial intelligence applied to nephrology has undergone a paradigm shift, driven by the rapid adoption of foundation models, large transformer architectures pre-trained on massive datasets, and then adapted to diverse downstream tasks. As introduced in Section 2.2, transformer attention mechanisms enable the transfer of knowledge to text, images, and, more recently, multimodal clinical data, enabling flexible fine-tuning or in-context learning with limited labeled nephrology datasets.

**Figure 16:**
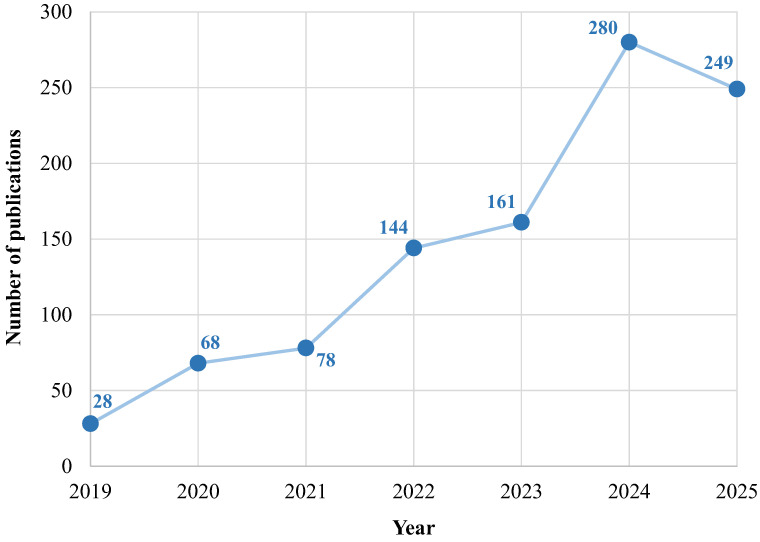
Number of publications per year on Artificial Intelligence (AI) applications related to the topic kidney disease and relevant therapies in the time interval 2019–2025. The research on the Scopus database used “Artificial Intelligence”, “Kidney Diseases”, and “Hemodialysis” as key search words, limited to English-language publications. Information updated on the 12th September 2025.

Used in a consistent way, these methods have seen a marked acceleration and have reached their peak impact over the last three years, as shown in Figure 16.

Specifically, this acceleration has led to a new generation of artificial intelligence systems capable of integrating heterogeneous sources of information, from laboratory data and imaging to clinical narratives, producing more robust and clinically meaningful predictions. Recent studies have shown that transformer-based models, compared with traditional machine learning models, can improve early identification of kidney disease, prediction of dialysis complications, and automated interpretation of renal pathology slides, while also providing attention maps and feature attribution that enhance interpretability for clinicians [216]. In addition, multimodal approaches combining imaging and clinical variables have demonstrated very high performance in differentiating diabetic nephropathy from non-diabetic renal disease and in improving pathologists’ confidence in membranous nephropathy diagnosis, compared to what has been performed in the past with less advanced models [217,218]. In this regard, recent reviews emphasized that multimodal AI is becoming a key tool to integrate imaging, laboratory data, and electronic health records in a single predictive framework, paving the way for automated, standardized, and explainable nephrology decision support [219]. Similarly, temporal attention architectures applied to hemodialysis time-series data have achieved AUC-ROC values close to 0.95 for intradialytic hypotension prediction, showing that such models can be deployed prospectively and offer real-time alerts [220].

Another emerging trend is the integration of large language models and conversational agents into nephrology practice. Notably, these models are increasingly being used to educate patients, answer common questions, support triage, and provide quick summaries of clinical guidelines for healthcare professionals, making access to information easier and improving patient engagement.

As evidence of this, early pilot studies have shown improvements in patient engagement and adherence to dietary and fluid intake recommendations [221,222], highlighting their potential role in participatory nephrology care. Their ability to perform reasoning, enhanced by the retrieval of clinical knowledge, lays the foundation for a future in which both patients and physicians will benefit from real-time, dialogue-based access to relevant medical information.

Alongside this shift towards transformers and, more specifically, foundation models, recent years have seen continued improvements in the performance of more classical machine learning approaches. Among these, random forest and gradient boosting models have been refined with larger, multicenter datasets, showing improved calibration and discrimination for predicting chronic kidney disease progression and acute kidney injury, in some cases reaching AUC-ROC values close to those of deep learning systems while remaining simpler and more transparent [223,224,225]. In addition, logistic regression-based tools such as the Kidney Failure Risk Equation (KFRE) have been validated in diverse populations, leading to recalibrated thresholds and improved applicability in non-North American cohorts. In regard to medical imaging, U-Net and its recent variants are still the backbone of kidney and tumor segmentation challenges, achieving composite Dice scores above 90% [226,227] and remaining competitive baselines against which transformer-based architectures are now compared. In addition, recent years have seen classical AI approaches applied in new domains of nephrology care, ranging from surrogate models for the prediction of the urea dialyzer clearance (R^2^ > 0.99) [228], to algorithms that estimate the most appropriate operational dialysis variables for each patient (R^2^ > 0.65) [229], or anticipate possible dialysis machine failures during sensor calibration tests [230].

Ultimately, these developments demonstrate that the evolution of AI in nephrology is occurring on multiple fronts: while foundation models expand the frontier of multimodal and interactive applications, classical machine learning continues to be optimized and remains highly relevant, especially where model simplicity, interpretability, and ease of clinical implementation are priorities.

### 3.6. Limitations and Challenges of Artificial Intelligence in Clinical Adoption

Although these studies highlight the remarkable performance of artificial intelligence models applied in nephrology, their results must be interpreted with caution. First, it should be emphasized that, despite some progress described in the previous section, most of the literature is still based on retrospective, single-center cohorts with limited demographic diversity, which makes the reported results highly context-dependent. This is because such datasets may lead to overfitting and generate models that are not transferable to heterogeneous populations or different healthcare infrastructures. Furthermore, frequent imbalances in case-control distributions and the lack of robust cross-validation or prospective testing reduce reliability, meaning that high technical performance does not necessarily translate into clinical benefit.

In addition, many deep learning and transformer-based models achieve outstanding predictive accuracy but remain “black boxes”, offering little transparency into the clinical rationale of their predictions. While methods already mentioned in Section 2.4, such as SHAP, LIME, and Grad-CAM, provide partial insights, their application is inconsistent and often difficult to interpret in real-time clinical settings.

Regulatory approval also remains a moving target, especially for generative models that change dynamically with continuous training, raising challenges for certification, monitoring, and liability. Along with this, such systems also face reproducibility issues and high computational cost, as well as ethical concerns regarding fairness, bias, and patient data privacy, particularly when datasets underrepresent minority or vulnerable populations.

Therefore, to move from proof-of-concept to real-world use, several hurdles need to be overcome, including external validation on large, multicenter datasets to ensure robustness and fairness, and improved interpretability to help clinicians understand the reasoning behind predictions. Regulatory pathways would also have to evolve to clarify approval and post-deployment monitoring, while hospital integration would need to be seamless and interoperable with EHRs and dialysis platforms, without adding burden to physicians. Finally, training programs for clinicians would be essential to foster trust and encourage appropriate adoption.

If these limitations are adequately addressed, AI could profoundly transform nephrology. Specifically, potential benefits include earlier and more accurate risk stratification, proactive detection of chronic kidney disease and acute kidney injury, real-time decision support for dialysis therapy, and optimized allocation of transplant organs. For patients, this could translate into longer survival, improved quality of life, and more equitable access to treatment. Conversely, uncritical adoption of such algorithms may lead to algorithmic bias, misclassification, reduced physician-patient communication, and excessive dependence on systems that lack transparency. It would, therefore, be crucial to adopt a balanced approach, leveraging AI as an augmentative tool rather than a substitute for clinical judgment, ensuring that technological progress does not come at the expense of empathy and human care.

## 4. Conclusions and Outlooks

This review has reported recent AI developments and advances in kidney disease diagnosis and hemodialysis treatment assistance. The innovations made by AI in this sector are noteworthy. Specifically, this review has focused on artificial intelligence research in the renal field conducted between 2019 and 2023. The papers discussed in the first part of this review have demonstrated how AI-based results support early prediction and determine the level of risk during the diagnosis of acute and chronic kidney disease, with a special focus on patients with previous pathologies, which are responsible for further deterioration of kidney function. The second part of this review has focused more specifically on patients with end-stage renal disease, covering artificial intelligence papers aimed at optimally predicting long-term complications of patients undergoing dialysis treatment and assisting nephrologists’ decision-making in managing therapy.

Most of these papers preferred the use of machine learning algorithms to deep learning models because of their greater simplicity of implementation and their greater interpretability, a key aspect for physicians who wish to understand the reasoning behind the models before adopting them into patient care. At the same time, DL approaches such as convolutional neural networks have shown great potential in medical image analysis, despite their greater complexity. Therefore, an important future challenge will be to balance interpretability and predictive power by making deep learning algorithms more transparent and accessible to healthcare professionals.

In addition, there is a significant disparity in the existing literature between papers dealing with AI techniques applied to prediction and therapy. While the first part of this review has highlighted a large number of studies on the role of AI in early prediction and risk determination during kidney disease diagnosis, the second part, which delves into end-stage renal disease and dialysis-related applications of AI, is relatively underrepresented. This imbalance underscores a crucial gap in current research, as end-stage renal disease patients make up a significant proportion of those in need of advanced medical support. Bridging this gap is essential to ensure that AI innovations comprehensively address the different stages of kidney disease and associated therapeutic challenges.

In conclusion, it is worth noting that true clinical trials, in which artificial intelligence models are directly integrated into real-world medical workflows as practical tools, have yet to be fully implemented. So far, these methods have been tested to prove their effectiveness compared to traditional approaches, but they have never been adopted as part of standard practices in clinical settings.

Contributing factors include clinicians’ skepticism toward automated systems, limited digital infrastructure in some healthcare environments, and the absence of clear regulatory frameworks. Overcoming these barriers will require targeted educational efforts to build trust among physicians, as well as investment in technological resources and institutional support. These measures would strengthen collaboration with healthcare providers and support the clinical validation of the models presented in this paper, which is essential for their integration into routine medical practice.

## Figures and Tables

**Table 1:** Formulas for the performance parameters of classification Machine Learning (ML) models are defined above.

Accuracy	$\frac{TP+TN}{FP+FN+TP+TN}$	(2)
Specificity	$\frac{TN}{FP+TN}$	(3)
Recall	$\frac{TP}{FN+TP}$	(4)
Precision	$\frac{TP}{TP+FP}$	(5)
F_β_-Score	$(1+\beta^{2})\cdot \frac{Precision\cdot TPR}{{(\beta }^{2}\cdot Precision)+TPR}$	(6)
FPR	$\frac{FP}{TN+FP}$	(7)
AP	$\frac{1}{m}\sum_{i=1}^{m}{P(R}_{i})$	(8)
mAP	$\frac{1}{m}\sum_{k=1}^{k=m}{AP}_{k}$	(9)
Dice coefficient	$\frac{2\cdot TP}{2\cdot TP+FP+FN}$	(10)
MCC	$\frac{TP\cdot TN-FP\cdot FN}{\sqrt{\left(TP+FP)(TP+FN)(TN+FP)(TN+FN\right)}}$	(11)

Where the symbol *k* in Equation (9) and the symbol *m* in Equations (8) and (9) represent the class and the total number of classes, respectively, while the expression ${P(R}_{i})$ in Equation (8) represents the Precision at each Recall point $R_{i}$ on the Area Under the Precision-Recall Curve (AUC-PR) curve.

**Table 2:** Formulas for the performance parameters of regression Machine Learning (ML) models.

R^2^ Score	$1-\frac{\sum_{i=1}^{n}(y_{i}-{\hat{y}}_{i}{)}^{2}}{\sum_{i=1}^{n}(y_{i}-{\bar{y}}_{i}{)}^{2}}$	(12)
MSE	$\frac{1}{n}\sum_{i=1}^{n}(y_{i}-{\hat{y}}_{i}{)}^{2}$	(13)
MAE	$\frac{1}{n}\sum_{i=1}^{n}\left|y_{i}\right.-\left.{\hat{y}}_{i}\right|$	(14)
RMSE	$\sqrt{\frac{1}{n}\sum_{i=1}^{n}(y_{i}-{\hat{y}}_{i}{)}^{2}}$	(15)
C-index	$\frac{Number\;of\;concordant\;pairs}{Total\;number\;of\;comparable\;pairs}$	(16)

Where the symbols *n*, $y_{i}$, and ${\hat{y}}_{i}$ in Equations (12)–(15) are the number of observations, the observed value of variable *y* for observation *i*, and the expected value of variable *y* for observation *i* according to the model, respectively. In Equation (16), the denominator identifies the pairs where one observation has experienced the event and the other has not, or if both have experienced the event but at different times. The numerator identifies the comparable pairs that are also concordant, that is, pairs that the prediction model correctly classifies according to the timing of the observed events.

**Table 3:** Comparative summary of the most frequently used Artificial Intelligence (AI) model families in nephrology.

Model Category	Models Used in Nephrology	Strengths	Limitations	Applications	Performance Metrics
Classification and Regression models	Logistic Regression, Ridge, LASSO, ElasticNet, SVM, Decision Tree, Random Forest, Extra Trees, kNN, Gradient Boosting	Linear models: simple, well-validated, highly interpretable, robust with small datasets; Tree-based/ boosting: capture non-linear relationships, handle missing data, high predictive accuracy, moderately interpretable	Linear models: cannot capture complex interactions; Tree-based/ boosting: risk of overfitting if not tuned, limited temporal modeling	Clinical risk prediction, hospitalization risk, EHR-based stratification	Linear models: AUC-ROC, Accuracy, C-index; Tree-based/ boosting: AUC-ROC, F_β_-Score
Feedforward Neural Networks	ANN, MLP	Capture complex non-linear patterns, flexible architectures	Require careful tuning, risk of overfitting with small data, less interpretable	CKD staging, prognosis prediction, comorbidity classification	Accuracy, AUC-ROC, F_β_-Score
Convolutional Neural Networks	CNN, U-Net, ResNet, DenseNet	State-of-the-art for image analysis, automatic feature extraction	Require large annotated datasets and resources, black-box nature	Kidney biopsy segmentation, CT/MRI classification	Dice coefficient, Accuracy, F_β_-Score
Transformer-based models	ViT, multimodal attention-based models	Strong performance on large-scale imaging, enable multimodal data fusion	Still emerging in nephrology, very data- and compute-intensive	Whole-slide image classification, multimodal pipelines	Accuracy, AUC-ROC, F_β_-Score
Recurrent Neural Networks	SimpleRNN, LSTM, GRU	Capture temporal dependencies, ideal for longitudinal monitoring	Computationally expensive, prone to overfitting with scarce data	Dialysis session forecasting, ICU time-series risk modeling	AUC-ROC, F_β_-Score, RMSE, MAE
Hybrid models	CNN-RNN, multimodal architectures	Combine multiple modalities, allow survival analysis and uncertainty quantification	Complex training and tuning, lower transparency	Multimodal risk prediction, personalized therapy optimization	C-index, AUC-ROC

**Table 5:** Literature overview on Machine Learning (ML) models developed for kidney disease detection.

Authors and Ref.	Year of Publication	Models	Dataset	Input Variables	Best Results	Aim
Akatsuka et al. [176]	2022	DenseNet model	24 glomerular WSIs of mice from Collagen Type IV Alpha 3 Chain Knock-Out (Col4a3 KO) mice; and from ADRamycin (ADR)-induced nephropathy mice	Structural and morphological features extracted by the model	Accuracy: 90%	Automatic evaluation of renal pathology through the detection of glomerular lesions in the renal tissues
Alikhan et al. [151]	2023	SACNN model + SOA algorithm	400 records from the ML repository of the University of California, Irvine, CA, USA	24 (demographic information, clinical variables, and laboratory data)	F_1_-Score: ≃96% Accuracy: ≃99%	Diagnosis and classification of CKD patients via IoT devices and cloud platforms
Al-Momani et al. [148]	2022	ANN model SVM model kNN model	400 records from the ML repository of the University of California, Irvine, CA, USA	13 (demographic information, clinical variables, and laboratory data)	Accuracy (ANN model): 99.20%	Diagnosis and classification of CKD based on patients’ data
Alsadi et al. [149]	2020	VGG-16 model VGG-19 model InceptionResNet-v2 model ResNet model NASNet model Inception-v3 model (transfer learning with pre-trained CNN for all six models)	900 EM images from native and transplant kidney biopsies from the University of Illinois Hospital & Health Sciences System, Chicago, IL, USA	Structural and morphological features extracted by the model	Accuracy (VGG-19 model): 78% F_1_-Score (VGG-19 model): ≃78%	Automatic evaluation of renal pathology by the identification of immune biological materials in renal biopsies
Altalbe et al. [159]	2023	CNN model	12,446 CT images from several undefined hospitals in Dhaka, Bangladesh	Structural and morphological features extracted by the model	F_1_-Score: 98.70% Accuracy: 99.20%	Diagnosis and classification of cysts, tumors, stones, and normal conditions using CT images
Asif et al. [165]	2023	StoneNet model	1799 CT images of 433 health patients or with renal stones from Elazıg Fethi Sekin City Hospital, Elazıg, Turkey	Structural and morphological features extracted by the model	Accuracy: 97.98% F_1_-Score: 97.87%	Detection of kidney stones in diseased patients for mobile devices
Bhaskar et al. [180]	2019	CNN model + SVM model	1000 saliva samples from 102 healthy and unhealthy patients from Vellore Institute of Technology (VIT), Tamil Nadu, India	Raw sensor signals extracted by the model	Accuracy: 98.04% R^2^: 97.99%	Detection of CKD patients based on urea concentration from saliva samples
Ginley et al. [169]	2021	DeepLab-v3 model DeepLab-v3 model + ResNet model	205 WSIs of fibrosis and glomerulosclerosis pathologies from five hospitals in Portugal, USA, and Republic of Korea	Structural and morphological features extracted by the model	MCC (DeepLab-v3 model + ResNet-50): 67% (IFTA), 89% (non-sclerotic glomeruli), 66% (glomerulosclerosis)	Automated detection of patients with IFTA, non-sclerotic glomeruli and glomerulosclerosis using WSIs
Gondim et al. [167]	2023	AI-based image classifier on Google AutoML Vision platform	252 renal neoplasm WSIs from four health institutions, USA	Structural and morphological features extracted by the model	AUC-PR: 93%	Detection and classification of patients with kidney neoplasms using WSIs
Granal et al. [152]	2022	BNN model	375 adult CKD patients from the Hopital Edouard Herriot, Lyon, France	25 (demographic data, previous pathologies, vital signs, medications, and nutritional parameters)	Accuracy: 74%	Estimation of potassium intake in CKD patients
Hao et al. [161]	2022	MN-Net model ResNet model	1199 WSIs of 1281 MN and healthy patients from the Second Hospital of Shanxi Medical University, Taiyuan, China; and from Shanxi Provincial People’s Hospital, Taiyuan, China	Structural and morphological features extracted by the model	Precision (MN-Net model): 95.34% F_1_-Score (MN-Net model): 97.62% Accuracy (MN-Net model): 95.69%	Diagnosis of MN patients through the detection and classification of glomeruli in renal pathological images
Hayashi [179]	2019	Re-RX model	1466 diabetic and pre-diabetic patients from the National Health And Nutrition Examination Survey (NHANES) database, USA	19 (demographic information, previous pathologies, clinical variables, patient habits, and metabolic variables)	Accuracy: 77.56% AUC-ROC: 75%	Detection of diabetic kidney diseases based on urinary Albumin-to-Creatinine Ratio (ACR) values
Jayapandian et al. [158]	2021	U-Net model	459 WSIs of 125 Minimal Change Disease (MCD) patients from the NEPTUNE digital pathology repository, North America, USA	Structural and morphological features extracted by the model	F_1_-Score: 94%	Diagnosis of CKD patients by automatic segmentation of histological structures of kidney tissue
Jhumka et al. [168]	2023	ResNet model	9527 CT images from several hospitals in Bangladesh, released by the online source Kaggle database, Google LLC, Mountain View, CA, USA; and from several hospitals in Turkey, released by the online source GitHub database, Microsoft, San Francisco, CA, USA	Structural and morphological features extracted by the model	F_1_-Score: ≃97% Accuracy: ≃97%	Detection and classification of patients with kidney cancers and stones using CT images
Khamparia et al. [147]	2020	Multilayer autoencoder + SoftMax probabilistic classifier	400 records from the ML repository of the University of California, Irvine, CA, USA	24 (demographic information, clinical variables, and laboratory data)	F_1_-Score: ≃100% Accuracy: ≃100%	Diagnosis of CKD among healthy and not healthy patients based on their data
Kumar et al. [160]	2023	Hybrid fuzzy-deep neural network	5617 CKD patients from Changhua Christian Hospital, Taichung, Taiwan	35 (demographic information, clinical variables, and previous pathologies)	Accuracy: 99.23%	Diagnosis of CKD based on patients’ data and images processing
Lassau et al. [153]	2019	ML model (unspecified)	4170 images (whose 787 of renal cortex) from forty-six public, private hospitals, and cancer centers, France	Structural and morphological features extracted by the model	Accuracy: ≃84% (>90% specifically on renal cortex)	Detection and characterization of tissue lesions through segmentation of several tissues, including the kidney cortex
Les et al. [155]	2020	U-Net model + EMT algorithm + SSA	1692 CT images of kidney lesions of 138 patients from the Military Institute of Medicine of Warsaw, Warsaw, Poland	Structural and morphological features extracted by the model	F_1_-Score: 89.30%	Detection of patients with kidney diseases by identification of kidney boundaries on CT images
Li et al. [164]	2022	3D U-Net model Res U-Net model SegNet model DeepLab-v3 model + UNETR model	260 renal CT scans from the First Affiliated Hospital of Guangzhou Medical University, Guangzhou, China	Structural and morphological features extracted by the model	Accuracy (Res U-Net): 99.96%	Detection of patients with kidney stones through segmentation of CT images
Lu et al. [174]	2022	BiT-S model BiT-M model	22,081 glomerular WSIs of 157 patients with kidney cancer from Vanderbilt University Pathology, Microbiology and Immunology, Nashville, TN, USA + 2340 glomerular images for external validation from an undefined center	Structural and morphological features extracted by the model	F_1_-Score (BiT-M model): 77.80% (internal validation) AUC-ROC (BiT-M model): 99.40% (external validation)	Detection and classification of patients with fine-grained Global GlomeruloSclerosis (GGS) using WSIs
Nan et al. [177]	2022	SegNet model + FISS loss function + UAAN model	400 WSIs of IgA nephropathy patients from the National Clinical Research Center of Kidney Diseases, Jinling, China + 165 histological images of colon tissue for FISS loss function validation from an undefined center	Structural and morphological features extracted by the model	Accuracy: 95.17%	Automatic detection of IgA nephropathy patients with fine-grained glomerular lesion using WSIs
Onthoni et al. [154]	2020	SSD Inception-v2 model	110 renal polycystic Contrast-enhanced Computed Tomography (CCT) images of 97 patients from the Picture Archiving and Communication System (PACS) of Linkou Chang Gung Memorial Hospital, Taoyuan, Taiwan	Structural and morphological features extracted by the model	mAP: 94%	Automatic detection and classification of patients affected by polycystic kidney disease
Parakh et al. [162]	2019	GrayNet-SB model ImageNet-SB model Random-SB model (transfer learning with pre-trained CNN for all three models)	535 renal polycystic CT images from a quaternary referral hospital, released by General Electric Healthcare company, Erlangen, Germany; and from Siemens Healthcare company, Little Chalfont, Buckinghamshire, UK	Structural and morphological features extracted by the model	AUC-ROC (GrayNet-SB model): 95.40% Accuracy (GrayNet-SB model): 95%	Detection of patients with stones within the urinary tract using CT scans
Pesce et al. [175]	2022	ANN feature-based model WVR model	2772 sclerotic and non-sclerotic glomeruli images of 26 biopsies from the Department of Emergency and Organ Transplantations (DETO) of Bari University Hospital, Bari, Italy	Textural and morphological features (150 extracted manually for ANN, extracted automatically by the model for WVR)	Accuracy (ANN feature-based model): 99% F_1_-Score (ANN feature-based model): 95.68%	Automatic detection of patients with glomerulosclerosis using histological images of renal biopsies
Pilia et al. [178]	2019	BNN model	71 ECGs of CKD patients from the simulation results of a modified Himeno et al. model + artificial data augmentation	5 (ECG features)	Generic test error (unspecified): 0.01 (for calcium), 0.25 (for potassium)	Estimation of extracellular calcium and potassium concentrations causing cardiovascular disease in CKD patients
Ram et al. [146]	2019	J48 DT model NB model SVM model LR model	Records (including CKD patients) from the ML repository of the University of California, Irvine, CA, USA	Demographic information, clinical variables, and laboratory data	Accuracy (J48 DT model): 99% (for CKD patients)	Detection of breast cancer, CKD patients, and dermatological diseases based on patients’ data
Sabanayagam et al. [181]	2020	CondenseNet model Risk Factors model CondenseNet model + Risk Factors model	12,970 retinal images of 6485 CKD and non-CKD patients from Singapore Epidemiology of Eye Diseases (SEED) Study, Singapore + 7470 images of 3735 CKD and non-CKD patients from Singapore Prospective Study Program (SP2), Singapore + 3076 images from 1538 CKD and non-CKD patients of Beijing Eye Study (BES), Beijing, China	5 (clinical variables, previous pathologies, demographic information) + structural and morphological features extracted by the model	AUC-ROC (CondenseNet model + Risk Factors model): 93.80% (SEED), 81% (SP2), 85.80% (BES)	Detection of CKD patients using patients’ retinal images and data
Senan et al. [145]	2021	SVM model kNN model DT model RF model	400 records of South Indian patients, released by the ML repository of the University of California, Irvine, CA, USA	24 (demographic information, clinical variables, and laboratory data)	F_1_-Score (RF model): ≃100% Accuracy (RF model): ≃100%	Diagnosis and classification of CKD patients based on their data
Subramanian et al. [166]	2023	CNN model Optimized CNN model	12,446 renal stones CT images from PACS of multiple hospitals, Bangladesh	Structural and morphological features extracted by the model	Accuracy (Optimized CNN model): ≃100%	Classification and detection of patients with cysts, renal tumors, and kidney stones using CT images
Tang et al. [171]	2022	Mask R-CNN model + DPCNN model	1727 US biopsy images of 251 patients with nephropathy from three hospitals affiliated with Taipei Medical University, Taipei, Taiwan + artificial data augmentation	Structural and morphological features extracted by the model	Dice coefficient: (Mask R-CNN): 94.90% F_1_-Score (DPCNN model): 81.90%	Detection of patients with IFTA and its severity using US images
Tsai et al. [156]	2022	ResNet model (pre-trained on the ImageNet database)	1599 pediatric US images from Taichung Veterans General Hospital, Taichung, Taiwan	Structural and morphological features extracted by the model	Accuracy: 92.90% AUC-ROC: 95.90%	Detection of pediatric renal anomalies using US images
Vashisth et al. [150]	2020	MLP model SVM model NB model	400 CKD patients, released by Apollo Hospitals, Tamil Nadu, India, released by the ML repository of the University of California, Irvine, CA, USA + online source Kaggle database, released by Google LLC, Mountain View, CA, USA	25 (demographic information, clinical variables, laboratory data, and previous pathologies)	Accuracy (MLP model): 92.50%	Detection of CKD based on patients’ data
Weis et al. [172]	2022	AlexNet model VGG model ResNet model DenseNet model SqueezeNet model InceptionNet model	23,575 renal biopsy images from the Institute of Pathology, Medical Faculty at Heidelberg University, Mannheim, Germany; and from the Institute of Pathology at Johannes Gutenberg University Mainz, Mainz, Germany	Structural and morphological features extracted by the model	Accuracy (ResNet model): 94.40%	Identification of CKD patients by recognition of glomerular morphologic patterns in renal biopsies
Yamaguchi et al. [157]	2020	ResNet model (pre-trained on the ImageNet database)	10,102 glomerular images of 293 WSIs from the University of Tokyo Hospital (UTH), Tokyo, Japan; from Tazuke Kofukai Medical Research Institute, Kitano Hospital (KH), Osaka, Japan; and from the University of Tsukuba Hospital, Tsukuba, Japan	Structural and morphological features extracted by the model	AUC-ROC: 98% (capillary collapse), 91% (fibrous crescent)	Detection of patients with renal pathologies by classification of glomerular images
Yi et al. [170]	2022	U-Net model + Mask R-CNN model	789 transplant biopsies of 616 patients from the Genomics of Chronic Allograft Rejection (GoCAR) Study, USA; and from the AUStralian Chronic Allograft Dysfunction (AUSCAD) Study, Australia	Structural and morphological features extracted by the model	Recall: 77% (mononuclear leukocyte infiltration), 85% (IFTA)	Detection of patients with IFTA, and mononuclear leukocyte infiltration in transplantation tissues
Yildirim et al. [163]	2021	ResNet model	1799 Non-Contrast Computed Tomography (NCCT) images of 433 healthy and unhealthy patients from Elazig Fethi Sekin City Hospital, Elazig, Turkey	Structural and morphological features extracted by the model	Accuracy: 96.82% F_1_-Score: 97%	Detection of patients with kidney stones using NCCT images
Zhang et al. [173]	2022	U-Net model + MANet model	1360 biopsy renal images from the Second Hospital of Shanxi Medical University, Taiyuan, China	Structural, fluorescent, and morphological features extracted by the model	Accuracy: 98% F_1_-Score: >95%	Detection of patients with glomerular diseases by segmentation and classification in immunofluorescence images
Zhang et al. [182]	2021	ResNet model (pre-trained on the ImageNet database)	115,344 ocular fundus images of 57,672 patients from China Consortium of Fundus Image Investigation (CC-FII), China; and from several hospitals in Beijing, Guangzhou, Chongqing and Tangshan, China	8 (clinical variables, previous pathologies, ad demographic information) + structural and morphological features extracted by the model	AUC-ROC: 93%	Detection of CKD patients using ocular fundus images and clinical data

**Table 6:** Literature overview on Machine Learning (ML) models developed as an assistant tool for nephrologists. Legend: n.a. = not available.

Authors and Ref.	Year of Publication	Models	Dataset	Input Variables	Best Results	Aim
Balamuthusamy et al. [192]	2023	XGBoost model RF model Multiple non-linear regression model	200 prevalent hemodialysis patients with an arteriovenous graft or AVF from the Plexus EHRs LLC platform, Dallas, TX, USA	20 (clinical variables, access intervention, previous surgeries, laboratory data, and demographic information)	Accuracy (XGBoost model): 86%	Real time risk stratification and re-intervention risk prediction on arteriovenous access of dialysis patients
Barbieri et al. [195]	2019	ANN model	766,000 dialysis sessions from the European Clinical Database (EuCliD) platform, released by NephroCare centers, Spain	Roughly 60 (demographic information, physiological variables, pre-dialysis data, and dialysis dose)	MAE: 0.23 kg (fluid volume removal), 7.3 bpm (heart rate), 9.3 mmHg (blood pressure), 0.13 (Kt/V)	Prediction of session-specific Kt/V, fluid volume removal, heart rate, and blood pressure of dialysis patients
Brito et al. [196]	2022	IBk model kStar model REPTree model RF model RandomTree model	2489 medical examinations from the Hospital Information System (HIS), released by Centro Hospitalar do Porto, Porto, Portugal	8 (demographic information, and dialysis dose)	Accuracy (RF model): 95.86%	Identification and classification of the serum creatinine values in patients undergoing peritoneal dialysis procedures
Cheng et al. [201]	2023	PD AI ChatBot on LINE Application	440 ESKD patients from the PD center of National Taiwan University Hospital, Taipei, Taiwan	Demographic information, nutritional data, and clinical variables	Degree of satisfaction: 90%	Self-management of patients undergoing peritoneal dialysis during the COVID-19 pandemic
Cho et al. [198]	2022	Cleerly platform	79 Coronary Computed Tomography Angiographies (CCTAs) of ESKD patients from two medical centers, Los Angeles, CA, USA	Structural and morphological features extracted by the model	Specificity: 91%	Identification of Atherosclerotic Plaque Characteristics (APC) in dialysis patients
Hong et al. [187]	2023	RF model GBM model Log. Reg. model (chosen among 18 pre-selected ML algorithms)	314,534 HD sessions of 3906 patients from Sichuan Provincial People’s Hospital, Chengdu, China	19 (demographic information, and clinical variables)	AUC-ROC (RF model): 81.20% F_1_-Score (RF model): 69.90% Accuracy (RF model): 74%	Identification of intradialytic hypotension in HD patients before initiating the treatment
Hong [194]	2021	CNN model	300 angiography images of 100 Maintenance HemoDialysis (MHD) patients from the Department of Gastroenterology of Tonglu First People’s Hospital, Hangzhou, Zhejiang, China	Clinical variables, adverse events, and morphological features extracted by the model	MSE: 0.005	Improvement of angiography images resolution performed on arteriovenous accesses of hemodialysis patients
Kanda et al. [199]	2020	Clustering model + SVM models Log. Reg. model DL model SVM model	79,860 ESKD patients from the Japanese Society for Dialysis Therapy (JSDT) Renal Data Registry, released by hospitals and clinics in Japan	20 (demographic information, previous pathologies, and clinical variables)	Accuracy (Clustering model + SVM models): 94.80% (1 year apart)	Early screening of hemodialysis patients at a high risk of death
Li et al. [184]	2022	bCOWOA-KELM model	1239 HD sessions of 156 patients from the First Affiliated Hospital of Wenzhou Medical University, Zhejiang, China + 6 ICU datasets from the ML repository of the University of California, Irvine, CA, USA	Demographic information, and clinical variables (36 for Wenzhou’s dataset, n.a. for California’s dataset)	Accuracy: 92.41% F_1_-Score: 93.36%	Prediction of IDH in hemodialysis patients using indices of blood routine tests
Mendoza-Pitti et al. [185]	2022	Log. Reg. model RF model MLP model XGBoost model	22,234 HD sessions of 299 patients from the Hospital Universitario Príncipe de Asturias, Madrid, Spain	20 (demographic information, previous pathologies, and clinical variables)	AUC-ROC (XGBoost model): 96.90% F_1_-Score (XGBoost model): 86% AUC-PR (XGBoost model): 94.50%	Prediction of the occurrence of IDH or non-IDH in HD patients at the beginning of the treatment
Mohammed et al. [186]	2023	RDLCDC-IDH model	1000 HD patients from undefined hospitals	Biometric data and clinical variables	F_1_-Score: 97.70% Accuracy: 97.70%	Diagnosis and classification of IDH in hemodialysis patients
Ota et al. [191]	2020	CNN model + LSTM model + GRU model	3994 AVF sounds of 20 patients from Gamagori Municipal Hospital, Gamagori-shi, Aichi, Japan	Acoustic features extracted by the model	AUC-ROC: 75–92% Accuracy: 82%	Diagnosis of stenosis through evaluation of AVF sounds
Othman et al. [189]	2022	MLP model kNN model SVM model DT model GBM model Bagging technique Voting technique RF model	6000 HD sessions of 215 adult patients from the Dialysis unit of El-Mowasah University Hospital, Alexandria, Egypt	12 (clinical variables, environmental information, dialysis characteristics, machine parameters, and nutritional data)	Accuracy (RF model): 98% F_1_-Score (GBM model): 92%	Early prediction of the most frequent hemodialysis complications
Qarajeh et al. [200]	2023	ChatGPT 3.5 ChatGPT 4 Bard AI Bing Chat	240 food items selected from the Mayo Clinic Renal Diet Handbook for CKD patients, USA	Nutritional data extracted by the model	Accuracy (ChatGPT 4): 81% (potassium) Precision: (Bard AI): ≃100% (phosphorus)	Categorization of foods into high or low potassium and high phosphorus content
Shih et al. [188]	2020	CNN model Transfer learning with a pre-trained VGG-16 + CNN model	80 physiological measures of each of 30 HD patients from the Division of Nephrology of Taichung Veterans General Hospital, Taichung, Taiwan	8 (clinical variables)	Accuracy (Transfer learning with a pre-trained VGG-16 + CNN model): 99%	Early warning of dialysis discomfort as hypotension, hypertension, and cramps in hemodialysis patients
Song et al. [190]	2023	ResNet model + ANN model	153 AVF sounds of 40 patients with mature AVF from several hospitals, Taiwan	Combination of input features typology based on Fourier transform and sample entropy	F_1_-Score: >90% Accuracy: >90%	Home detection of AVF stenosis using audio recordings
Tan et al. [197]	2022	YOLACT model Mask R-CNN model	1385 LUS images of 76 patients from the Division of Renal Medicine of Khoo Teck Puat Hospital, Singapore + Microsoft Common Objects in COntext (COCO) data for model pre-training	Demographic characteristics, vital signs, clinical variables, and bioimpedance + structural and morphological features extracted by the model	Accuracy: ≃69% Recall: 83.30% Precision: 65.30%	Identification of fluids overload in hemodialysis patients using US images
Zhang et al. [193]	2021	Transfer learning with a pre-trained model from Amazon SageMaker platform + CNN model	1341 arteriovenous access images from 20 dialysis clinics across six States, USA	Structural and morphological features extracted by the model	AUC-ROC: 96%	Classification of arteriovenous access aneurysms in hemodialysis patients

**Table 7:** Literature overview on Machine Learning (ML) models developed as a tool to predict long-term complications in dialysis patients.

Authors and Ref.	Year of Publication	Models	Dataset	Input Variables	Best Results	Aim
Díez-Sanmartín et al. [205]	2023	Clustering models + XGBoost model + FCDR algorithm	44,663 adults awaiting transplantation from the Organ Procurement and Transplantation Network (OPTN) medical dataset, USA	7 (sociodemographic information)	AUC-ROC: 99.08%	Prediction of survival time for dialyzed patients on the kidney transplant waiting list
Garbelli et al. [211]	2022	BINCM model CORN model REGM model	46,292 blood flow measurements in AVFs of 5940 HD patients from Czech Republic, Portugal, Slovakia, and Spain	49 (demographic information, clinical variables, and machine parameters)	MAE (BINCM model): 0.27	Prediction of AVF flow level in hemodialysis patients during the treatment
Gotta et al. [208]	2021	RF model	363 HD patients from several DaVita Kidney Care centers, USA	12 (demographic information, laboratory data, and dialysis characteristics)	Accuracy: 81%	Identification of key predictors for 5-year mortality in pediatric and young adult patients undergoing hemodialysis
Kong et al. [215]	2021	SVM model + RF model + kNN model + Log. Reg. model	23,992 PD patients from the Hospital Quality Monitoring System (HQMS), China	15 (demographic information, clinical variables, reason for admission, and previous pathologies)	AUC-ROC: 75.70% Accuracy: 69.50%	Prediction of the p-LOS risk for peritoneal patients
Liao et al. [210]	2023	kNN model Gaussian NB model Log. Reg. model SVM model MLP model DT model RF model AdaBoost model GBM model LGBM model Vote classifiers with tests on all models	242 MHD patients from Wenjiang Hemodialysis Center of the Department of Nephrology in West China Hospital, Sichuan University, Chengdu, China	Demographic information, body measurement results, and laboratory data (4 for women, 3 for men)	AUC-ROC: 87.40% (for men), 77.69% (for women) F_1_-Score: 77.32% (for men), 78.04% (for women) For men: vote classifier composed by Log. Reg. model + AdaBoost model + LGBM model For women: SVM model	Early identification of simple sarcopenia for MHD patients
Monaghan et al. [212]	2021	XGBoost model	40,490 HD patients from a national network of dialysis clinics, MA, USA	81 (laboratory data, and clinical variables)	AUC-ROC: 68%	Prediction of the risk of HD patients having a COVID-19 infection within three days
Noh et al. [206]	2020	DT model Bagging model RF model SVM model ANN model Log. Reg. model LSTM model + Autoencoder model Ridge model LASSO model Survival Tree model	1730 PD patients from the Clinical Research Center for ESRD dataset, released by 36 general and teaching hospitals, Republic of Korea	23 (demographic information, clinical variables, previous pathologies, and laboratory data)	AUC-ROC (LSTM model + Autoencoder model): 85.80% C-index (Survival Tree model): 76.90%	Prediction of the mortality risk in peritoneal dialysis patients
Ohara et al. [209]	2021	AISACS model	298 HD sessions of 16 patients from Kobayashi Medical Clinic, Okayama, Japan + 7937 HD sessions of 211 patients from Shigei Medical Research Hospital, Okayama, Japan	4 (blood parameters)	Accuracy: up to 87%	Management of patient anemia based on blood parameters of hemodialysis patients
Siga et al. [207]	2020	Optimized BNN model Log. Reg. model	4915 HD patients from the prospective cohort study Photo-Graph-v3, released by a multicenter sample of nephrologists, France	14 (demographic information, laboratory data, and dialysis characteristics)	AUC-ROC (Optimized BNN model): 78%	Prediction of all-cause mortality in hemodialysis patients
Tang et al. [213]	2023	LGBM model CatBoost model RF model XGBoost model	433 HD patients from Taipei VGH, Taipei, Taiwan	29 (demographic information, laboratory data, previous diseases, and vaccination information)	F_1_-Score (LGBM model): 95%	Prediction of the survival impact for partial COVID-19 vaccination in hemodialysis patients
Wu et al. [214]	2020	CART model RF model GBM model	22,859 PD patients from the HQMS, China	34 (demographic information, previous diseases, and clinical data)	AUC-ROC (RF model): 75.60%	Prediction of the p-LOS risk in peritoneal dialysis patients

## Data Availability

The original contributions presented in the study are included in the article, further inquiries can be directed to the corresponding author.

## Abbreviations

ACO	Ant Colony-based Optimization
ACR	Albumin-to-Creatinine Ratio
AdaBoost	Adaptive Boosting
ADAM	ADAptive Moment estimation
ADR	ADRamycin
AHDCNN	Adaptive Hybridized Deep Convolutional Neural Network
AI	Artificial Intelligence
AISACS	Artificial Intelligence Supported Anemia Control System
AKI	Acute Kidney Injury
AlexNet	Alex Network
ANN	Artificial Neural Network
AP	Average Precision
APC	Atherosclerotic Plaque Characteristics
AUC-PR	Area Under the Precision-Recall Curve
AUC-ROC	Area Under the Receiver Operating Characteristic Curve
AUSCAD	AUStralian Chronic Allograft Dysfunction
AVF	ArterioVenous Fistula
bCO	binary COvariance
BDF	Bahrain Defence Force
BES	Beijing Eye Study
BIDMC	Beth Israel Deaconess Medical Center
BINCM	BINary Classifier Model
BiT-M	Big Transfer-Medium
BiT-S	Big Transfer-Small
BNN	Bayesian Neural Network
BoW	Bag-of-Words
CART	Classification And Regression Tree
CatBoost	Categorical Boosting
CC-FII	China Consortium of Fundus Image Investigation
ccRCC	clear-cell Renal-Cell Carcinoma
CCT	Contrast-enhanced Computed Tomography
CCTA	Coronary Computed Tomography Angiography
ChatGPT	Chat Generative Pre-trained Transformer
C-index	Concordance index
CIT	Conditional Inference Tree
CKD	Chronic Kidney Disease
CKDu	Chronic Kidney Disease of unknown etiology
CNN	Convolutional Neural Network
COCO	Common Objects in COntext
Col4a3 KO	Collagen Type IV Alpha 3 Chain Knock-Out
CondenseNet	Condensed Network
CORN	Consistent Ordinal Regression Network
CPTAC	Clinical Proteomic Tumor Analysis Consortium
CSA-AKI	Cardiac Surgery-Associated Acute Kidney Injury
CT	Computational Tomography
DCDLT	Donation after Cardiac Death Liver Transplantation
DDLT	Deceased-Donor Liver Transplantation
DEMATEL	Decision Making Trial And Evaluation Laboratory
DenseNet	Dense Network
DETO	Department of Emergency and Organ Transplantations
DFS	Density-based Feature Selection
DINO-ViT	DIstillation with NO labels-Vision Transformer
DISPO	Dynamic Integrative System for Predicting Outcome
DL	Deep Learning
DNN	Deep Neural Network
DPCNN	Dual-Path Convolutional Neural Network
DS-GBT	Deep Support-Gradient Boosting Trees
DT	Decision Tree
ECG	ElectroCardioGram
eGFR	estimated Glomerular Filtration Rate
EHR	Electronic Health Record
eICU	electronic Intensive Care Unit
EM	Electron Microscopy
EMT	Extended Maxima Transform
eqU	equilibrated post-dialysis blood Urea
ESKD	End-Stage Kidney Disease
EuCliD	European Clinical Database
Extra Trees	Extremely Randomized Trees
FCDR	False Clustering Discovery Reduction
FDA	Fisher Discriminant Analysis
FEMH	Far Eastern Memorial Hospital
FISS	Focal Instance Structural Similarity
FMC	Fresenius Medical Care
FN	False Negative
FNN	Feedforward Neural Network
FP	False Positive
FPR	False Positive Rate
GAM	Generalised Additive Model
GAN	Generative Adversarial Network
GBDT	Gradient Boosting Decision Tree
GBM	Gradient Boosting Machine
GCKD	German Chronic Kidney Disease
GGS	Global GlomeruloSclerosis
GMM	Gaussian Mixture Model
GoCAR	Genomics of Chronic Allograft Rejection
Grad-CAM	Gradient-weighted Class Activation Mapping
GRNN	Generalised Regression Neural Network
GRU	Gated Recurrent Unit
GSA	Gravitational Search Algorithm
HD	HemoDialysis
HDF	HemoDiaFiltration
HERON	Healthcare Enterprise Repository for Ontological Narration
HF	HemoFiltration
HGSAPSO	Hybrid Gravitational Search Algorithm and Particle Swarm Optimization
HIS	Hospital Information System
HQMS	Hospital Quality Monitoring System
ICU	Intensive Care Unit
IDEA	Intraoperative and Data Embedded Analytics
IDH	IntraDialytic Hypotension
IDS	Intelligent Dosing System
IFTA	Interstitial Fibrosis and Tubular Atrophy
IgAN	Immunoglobulin A Nephropathy
InceptionNet	Inception Network
InceptionResNet-v2	Inception-Residual Network version 2
IoT	Internet of Things
JSDT	Japanese Society for Dialysis Therapy
KD	Kidney Disease
KDQOL	Kidney Disease Quality Of Life
KELM	Kernel Extreme Learning Machine
KFRE	Kidney Failure Risk Equation
KH	Kitano Hospital
kNN	k-Nearest Neighbors
LASSO	Least Absolute Shrinkage and Selection Operator
LDA	Linear Discriminant Analysis
LGBM	Light Gradient Boosting Machine
LIME	Local Interpretable Model-agnostic Explanations
LLC	Limited Liability Company
LLM	Large Language Model
Log. Reg.	Logistic Regression
LogitBoost	Logistic Boosting
LR	Linear Regression
LSTM	Long Short Term Memory
LUS	Lung UltraSound
MAE	Mean Absolute Error
MANet	Multiple-Attention Network
mAP	mean Average Precision
Mask R-CNN	Mask Region-based Convolutional Neural Network
MCC	Matthews Correlation Coefficient
MCD	Minimal Change Disease
MHD	Maintenance HemoDialysis
MIL	Multiple Instance Learning
MIMIC-III/IV	Medical Information Mart for Intensive Care III/IV
ML	Machine Learning
MLP	MultiLayer Perceptron
MMDLM	MultiModal Deep Learning Model
MN	Membranous Nephropathy
MN-Net	Membranous Nephropathy Network
MRI	Magnetic Resonance Image
MSE	Mean Squared Error
NASNet	Neural Architecture Search Network
NB	Naive Bayes
NCCT	Non-Contrast Computed Tomography
NHANES	National Health And Nutrition Examination Survey
NHISS	National Health Insurance Sharing Service
NHS	National Health Service
NIH	National Institutes of Health
NLP	Natural Language Processing
NMR	Nuclear Magnetic Resonance
NN	Neural Network
OPTN	Organ Procurement and Transplantation Network
PACS	Picture Archiving and Communication System
PCNL	PerCutaneous NephroLithotomy
p-LOS	prolonged Length Of Stay
PNN	Probabilistic Neural Network
post-EVAR	post-EndoVascular Aneurysm Repair
PSO	Particle Swarm Optimization
QDA	Quadratic Discriminant Analysis
RBFNN	Radial Basis Function Neural Network
RDLCDC	Robust Deep Learning-based Clinical Data Classification
REPTree	Reduced Error Pruning Tree
Re-RX	Recursive Rule eXtraction
ResNet	Residual Network
RF	Random Forest
RMDS	Renal Mass Diagnostic System
RMSE	Root Mean Squared Error
RMSPROP	Root Mean Square PROPagation
RNN	Recurrent Neural Network
RWTH	Rheinisch-Westfälische Technische Hochschule
SACNN	Self-Attention Convolutional Neural Network
SB	Surface-Based
SEED	Singapore Epidemiology of Eye Diseases
Seq2Seq	Sequence-To-Sequence
SFS	Sequential Forward Selection
SGDM	Stochastic Gradient Descent with Momentum
SHAP	SHapley Additive exPlanations
SimpleRNN	Simple Recurrent Neural Network
SOA	Season Optimization Algorithm
SP2	Singapore Prospective Study Program
SqueezeNet	Squeeze Network
SRI	Solute Removal Index
SRTR	Scientific Registry of Transplant Recipients
SRU	Simple Recurrent Unit
SSA	Slice Scanning Algorithm
SSD	Single Shot Detector
SSO	Sparrow Search Optimization
SVM	Support Vector Machine
TAN	Tree Augmented Naive
TCGA	The Cancer Genome Atlas
TN	True Negative
TP	True Positive
TPR	True Positive Rate
UAAN	Uncertainty-Aided Apportionment Network
U-Net	U-Network
UNETR	U-NEt TRansformer
US	UltraSound
UTH	University of Tokyo Hospital
VA	Veterans Affairs
VAE	Variational AutoEncoder
VB-MrFo-Net	V Bottleneck Multi-resolution and Focus-organ Network
VGG-16/19	Visual Geometry Group 16/19
VGH	Veterans General Hospital
VIT	Vellore Institute of Technology
ViT	Vision Transformer
WOA	Whale Optimization Algorithm
WSI	Whole-Slide Image
WVR	Watson Visual Recognition
XAI	eXplainable AI
XGBoost	eXtreme Gradient Boosting
